# CREB Regulates Cisplatin Resistance by Targeting TNKS and KDM6A in NSCLC cell-Derived Tumor Spheroid

**DOI:** 10.7150/ijbs.109419

**Published:** 2025-07-28

**Authors:** Ji Hae Lee, Youngjoo Kwon, Kyungsil Yoon

**Affiliations:** 1 Cancer Metastasis Branch, National Cancer Center, Goyang 10408, Korea.; 2 College of Pharmacy, Graduate School of Pharmaceutical Sciences, Ewha Womans University, Seoul 03760, Korea.

**Keywords:** NSCLC, cisplatin, resistance, CREB, TNKS, KDM6A

## Abstract

Platinum-based chemotherapy is the standard treatment for advanced non-small cell lung cancer (NSCLC); however, innate and acquired resistance is a major obstacle. To determine the transcriptional regulators of resistance, we first classified three-dimensional tumor spheroids derived from 11 NSCLC cell lines into cisplatin-sensitive or -resistant groups based on their cisplatin sensitivity and selected signature genes that were differentially altered between the groups. Using reverse engineering methods and functional validation, cAMP response element-binding protein 1 (CREB) was identified as a major regulator of cisplatin resistance. Among the putative target genes of CREB responsible for cisplatin resistance, cisplatin treatment significantly decreased the occupancy of CREB in the regulatory regions of *TNKS* and *KDM6A* in cisplatin-sensitive cells, but not in resistant cells, resulting in decreased expression of these protein in the sensitive group. Furthermore, CREB knockdown led to increased sensitivity to cisplatin with reduced levels of TNKS and KDM6A in both cisplatin-resistant tumor spheroids and tumors in a xenograft mouse model. In conclusion, our study delineates the role of CREB in cisplatin resistance and suggests that CREB inhibition is a potential therapeutic strategy for cisplatin-resistant NSCLCs.

## Introduction

Lung cancer is a deadly disease that claims more lives than any other cancer worldwide 1. Non-small cell lung cancer (NSCLC) accounts for approximately 85% of all lung cancers. Most patients with NSCLC present with regional (22%) or distant metastases (44%) at the time of diagnosis, and their 5-year survival rates are 34% and 7%, respectively 2. Although numerous targeted and immunotherapeutic agents have been successfully developed, platinum-based chemotherapy remains a treatment option for patients with advanced-stage NSCLCs, especially those who are not eligible for other treatment regimens. In addition, platinum-based chemotherapy is used in combination with surgery as adjuvant therapy and immunotherapy for the treatment of NSCLC patients 3-7.

Cisplatin, a first-generation platinum-based anticancer drug, is used to treat various solid tumors 8, however, intrinsic and/or acquired resistance to cisplatin restricts its therapeutic efficacy 9. Hence, the identification of the key factors that contribute to cisplatin resistance is necessary to enhance the effectiveness of cisplatin-based chemotherapy.

Reverse engineering is an approach for reconstructing regulatory networks from gene expression profiles by identifying interactions between genes and regulatory elements and prioritizing key transcription regulators within these networks. This method is widely used to infer gene regulatory networks and provides insights into how genes interact within complex networks to influence cellular phenotypes and behavior under various conditions 10, 11.

Cyclic AMP response element-binding protein 1 (CREB) is a basic leucine zipper (bZIP) transcription factor that binds to a conserved cAMP response element (CRE), 5'-TGACGTCA-3', in the regulatory region of genes to regulate gene expression involved in metabolism, neurotransmission, cell cycle, and cell survival 12-14. CREB is activated by phosphorylation at serine 133 (Ser133) in response to various serine-threonine kinases, such as protein kinase A (PKA) 12, mitogen-activated protein kinase (MAPK) 15, 16, calcium/calmodulin-dependent protein kinase (CaMK) 17, 18, and protein kinase B (Akt) 19. The transcriptional activity of CREB is modulated by coactivators, including CREB-binding protein (CBP) and CREB-regulated transcriptional coactivators (CRTCs). CBP acetyltransferase specifically interacts with the Ser133 of CREB 20-23. Binding of CRTCs to the bZIP domain of CREB enhances the occupancy of target gene promoters 24, 25. The dysregulation of CREB contributes to the development of various diseases, including metabolic disorders 26, neurodegenerative disorders 27, and different types of cancers, such as breast 28, ovarian 29, and lung cancers 30. The levels of *CREB* mRNA, as well as CREB and pCREB proteins were higher in NSCLC specimens than in adjacent normal lung 30.

Recently, three-dimensional (3D) cell culture has emerged as an approach that mimics the physiological environment of *in vivo* tumors better than traditional two-dimensional (2D) cell culture 31. Tumor spheroids are self-assembled 3D aggregates of cancer cells that often exhibit similar nutrient, oxygen, and pH levels to *in vivo* tumors 32-36. This gradient arises because the outer cell layer in the spheroid has greater access to nutrients and oxygen than the inner cells, which can lead to differences in cell viability, proliferation, and gene expression 37. This natural gradient in tumor spheroids leads to the development of a hypoxic microenvironment, which increases drug resistance in cancer cells 38. Physical cell-cell and cell-extracellular matrix (ECM) interactions also contribute to resistance to chemotherapy 39-41. Thus, tumor spheroids provide a useful model for studying drug resistance *in vitro*, which will help develop new therapeutic targets and strategies for overcoming resistance.

In this study, we conducted a transcriptional regulatory network analysis using RNA-seq data obtained from cisplatin-resistant and -sensitive tumor spheroids derived from NSCLC cell lines to identify key factors associated with chemoresistance. Our findings highlight CREB as a master regulator of cisplatin resistance and suggest that inhibiting CREB during cisplatin treatment could serve as a novel therapeutic strategy for treating cisplatin-resistant NSCLCs.

## Materials and Methods

### Cell line and cell cultures

A549, NCI-H1299, NCI-H358, NCI-H1975, Calu-6, NCI-H460, NCI-H522, NCI-H596, NCI-H23, and NCI-H1703 cell lines were obtained from the American Type Culture Collection (USA), while HCC2279 (KCLB No. 72279) was purchased from the Korea Cell Line Bank (Korea). All cancer cell lines were maintained at 37°C with 5% CO₂ in RPMI-1640 medium supplemented with penicillin (100 units/ml), streptomycin (100 µg/ml), and 10% fetal bovine serum. To produce tumor spheroids, cells were seeded in 96-well round-bottom plates (34896, SPL, Korea) at the following densities and centrifuged at 1000 rpm for 10 minutes: NCI-H358 and NCI-H596 at 6 × 10⁴ cells/well; A549, HCC2279, and NCI-H1975 at 5 × 10⁴ cells/well; NCI-H460 and Calu-6 at 2 × 10⁴ cells/well; NCI-H1299, NCI-H23, and NCI-H1703 at 3 × 10⁴ cells/well; NCI-H522 at 4 × 10⁴ cells/well. Tumor spheroids were cultured in RPMI-1640 medium with Matrigel (BD Biosciences, USA): 0.5% Matrigel for A549, NCI-H1299, NCI-H358, NCI-H460, NCI-H522, NCI-H596, NCI-H23, and NCI-H1703, and 2% Matrigel for NCI-H1975, HCC2279, and Calu-6. Cisplatin (Dong-A Pharmaceutical Co. Ltd, Korea) treatment was applied three days after tumor spheroid formation.

### RNA interference

According to the manufacturer's instructions, cells were transfected with siRNA using Lipofectamine RNAiMAX (Invitrogen, USA). The siRNA sequences used were as follows: ATF6 (#1 5'-CAGAGAACUGUCUCGUACU-3', #2 5'-CAGUGUUACCUGGUGUAGA-3'), CREB (#1 5'-CUGCAAACAUUAACCAUGA-3', #2 5'-UCAAGGAGGCCUUCCUACA-3'), CTCF (#1 5'-CUGGUAUGGUCAAGCUUGU-3', #2 5'-AGUUAGCUAUCAGACUCUA-3'), HIVEP1 (#1 5'-CUCCUUGUGUAAGACAGUA-3', #2 5'-CUGAUGAGAGACAGCAUGA-3'), GTF2IRD1 (#1 5'-CGUACUCCAAGUUUCUGAU-3', #2 5'-CCUGGAGAGGAUUCUUGCU-3'), FoxJ3 (#1 5'-CAGUGUUAAUUGGUCAGAU-3', #2 5'-ACUGAUGUAGGAAACUGAA-3'), PBX3 (#1 5'-GAGUGUUGUAUAUAGUGUA-3', #2 5'-CAGAACUGGAGAAAUAUGA-3'), TFDP2 (#1 5'-GGCUCUGAACUCUACCAUU-3', #2 5'-GCAGAAGUGGCCUUAGCAA-3'), TCF7L2 (#1 5'-AGAGAAGAGCAAGCGAAAUA-3', #2 5'-UAGCUGAGUGCACGUUGAAAG-3'), MLLT10 (#1 5'-GGACCGUGGUUUUGCAGGA-3', #2 5'-GAGAACCCGCUGGUUUAUU-3'), BRSK2 (#1 5'-CAUCCGCAUCGCAGACUUU-3', #2 5'-CUUCGACGAUGACAACUUG-3'), ASAP1 (#1 5'-CCACCUGGCUUUCAACCAA-3', #2 5'-CCAUUUGGUUGACUUCCUU-3'), FGD6 (#1 5'-GCACAGGACUUAGUCAAUU-3', #2 5'-GCACUGGACUGAACAACAA-3'), RALGPS2 (#1 5'-GCAGGCAAGCAGUGUCAAU-3', #2 5'-GCUGCUUCCAGAGAAGAUU-3'), TBL1XR1 (#1 5'-GCAGCAUAAAGGCCCUAUA-3', #2 5'-GCCUGAUGUAGUACAAACA-3'), PDS5A (#1 5'-UUCUUCCUCAGGAACCCCA-3'), TNKS (#1 5'-CUACAACAGAGUUCGAAUA-3', #2 5'-GUGUGUAAAUGGAACAGAU-3'), MAP4 (#1 5'-GAGGAGAUGUCAAGAUUGA-3', #2 5'-GCCCACAGAAACAGAUGUA-3'), KDM6A (#1 5'-CUCAGAUAGUGAAUCUACA-3', #2 5'-CUGGUAUUCAHAAUCAGAA-3'), TBL1X (#1 5'-GGAAAUAGAUGGAGAGGUU-3', #2 5'-GCCUUGAAAUGGAACCGAA-3'), SRGAP1 (#1 5'-GCAAAGACCAUGCAACCUU-3', #2 5'-GCAAACUCCUCCUGACUUU-3'), and STAT3 (#1 5'-CAGCAAAAAGUUUCCUACA-3', #2 5'-UGUUCUCUGAGACCCAUGA-3'). A non-targeting control siRNA was obtained from Bioneer (Korea). Cells were seeded in a 6-well plate and transfected with 20 nM siRNA. After transfection for 6 h, cells were reseeded into a 96-well round-bottom plate.

### Quantitative PCR (qPCR)

Total RNA was isolated from tumor spheroids using the RNeasy Mini Kit (Qiagen, Germany), and cDNA was synthesized with a cDNA synthesis kit (Dyne Bio, Korea). qPCR was performed using SFC green qPCR master mix (BioFACT, Korea) on the LightCycler 96 Real-Time PCR System (Roche, Germany). Primers used for qPCR were as follows: *CREB* (Forward: 5′-TGGAGTCATTCAGGCGGC-3, Reverse: 5′-AGTTGAAATCTGAACTGTTTGGAC-3); *TNKS* (Forward: 5′-AGAGTACCTGCTACACCACGGT-3, Reverse: 5′-AGTCCGCCACATTGACAGAAGC-3); *KDM6A* (Forward: 5′-TACAGGCTCAGTTGTGTAACCT-3, Reverse: 5′-CTGCGGGAATTGGTAGGCTC-3); *GAPDH* (Forward: 5′-TGCACCACCAACTGCTTAGC-3, Reverse: 5′-GGCATGGACTGTGGTCATGAG-3); *HPRT* (Forward: 5′-GCTATAAATTCTTTGCTGACCTGCTG-3, Reverse: 5′-AATTACTTTTATGTCCCCTGTTGACTGG-3). *GAPDH* and *HPRT* were used as internal controls for normalization. Relative gene expression was quantified using the 2^-ΔΔCt^ method.

### Viability assay and live/dead cell staining of tumor spheroids

Tumor spheroid viability was measured using the CellTiter-Glo® 3D cell viability assay (Promega, USA) according to the manufacturer's protocol, and luminescence signals were detected with the GloMax-Multi Detection System (Promega, USA). For live/dead cell staining, tumor spheroids were incubated with 1 µM calcein-acetoxymethyl ester (AM) and 10 µM ethidium homodimer-1 (EthD-1) for 20 min at 37°C using the LIVE/DEAD™ Viability/Cytotoxicity Kit (Thermo Scientific, USA). Image acquisition was performed with the Operetta High Content Screening System (PerkinElmer, USA) and analyzed using Harmony 3.5.2 software.

### Luciferase reporter assay

The pGL4.29 vector containing a cAMP response element (CRE) was purchased from Promega. DNA fragments corresponding to the transcriptional regulatory regions of *TNKS* and *KDM6A*, including wild-type and mutant forms carrying single or double mutations in CREB binding sites, were synthesized and subcloned into the pGL3-basic vector. A schematic illustration of the wild-type and mutant constructs used in the luciferase assays is presented in Figure 6E. CREB binding sites were mutated as follows: BS1, CCGGTTGACGGT changed to CCGGTAGTGGGTA; BS2, CCACGTCA changed to CCAAATTC; BS3, TCACGTGA changed to TCAAATTC; and BS4, TGACGTGA changed to AGTGGTGA. Cells were seeded in 48-well plates and co-transfected with either the pGL4.29-CRE reporter or pGL3-based constructs harboring wild-type or mutant regulatory regions of *TNKS* or *KDM6A*, along with the pRL-CMV renilla luciferase control vector, using Lipofectamine 2000 (Invitrogen). After 24 h of transfection, cells were treated with cisplatin for an additional 24 h. Cells were then lysed in passive lysis buffer, and firefly and renilla luciferase activities were measured using the Dual-Glo luciferase assay system (Promega, USA) and GloMax-Multi Detection System (Promega, USA). Firefly luciferase activity was normalized to renilla luciferase activity to control for transfection efficiency.

### Western blotting

Cells were lysed with radio immunoprecipitation assay (RIPA) buffer (89900, Thermo Scientific, USA) supplemented with a protease inhibitor cocktail, phosphatase inhibitor (Calbiochem, USA), and phenylmethylsulfonyl fluoride (PMSF). Protein concentrations were measured using a bicinchoninic acid (BCA) protein assay kit (Thermo Scientific, USA). Proteins were separated by 8-12% sodium dodecyl sulfate-polyacrylamide gel electrophoresis (SDS-PAGE) and transferred onto a polyvinylidene fluoride (PVDF) membrane (Millipore, USA). Antibodies against CREB (#9197), phospho-CREB (#9198), KDM6A (#33510), BRSK2 (#5460), STAT3 (#9139), phospho-STAT3 (#9145), cleaved caspase-3 (#9661), cleaved caspase-7 (#9491), cleaved PARP (#9541), phospho-Akt (#9275), phospho-Chk1 (#2348), Chk1 (#2360), phospho-PKA C (#4781), PKA C-α (#4782), phospho-JNK (#4668), JNK (#9252), phospho-p38 (#4511), p38 (#8690), phospho-Erk1/2 (#4370), GAPDH (#2118), and β-actin (#4970) were obtained from Cell Signaling. An antibody against TNKS (GTX117417) and phospho-ATR (GTX128145) were purchased from GeneTex. Secondary antibodies, goat anti-mouse IgG HRP (SA001-500) and goat anti-rabbit IgG HRP (SA002-500), were purchased from GenDEPOT.

### Chromatin immunoprecipitation (ChIP) assay

ChIP assay was performed according to protocol provided from Cell signaling. Briefly, cells grown to 70% confluence in a 150 mm plate were treated with vehicle and cisplatin for 24 h. Extra plates of cells in each treatment were prepared to determine cell number. Formaldehyde (37%) was added directly to the cell culture plate to a final concentration of 1% in 20 ml of medium. Fixation was carried out at room temperature for 10 min and stopped by adding glycine to a final concentration of 0.125 M for 5 min. The cells were rinsed with cold PBS and collected by centrifugation at 500 x g for 5 min at 4°C. The 2 × 10^7^ cells were resuspended in buffer A (#7006, Cell Signaling) and incubated on ice for 10 min. The nuclei were collected by centrifugation at 2000 x g for 5 min at 4°C and then resuspended in buffer B (#7007, Cell Signaling). Micrococcal Nuclease (#10011, Cell Signaling) was added at 2.5 μl per 2 × 10^7^ cells and incubated 37°C for 20 min to digest chromatin. Pellet was resuspended with ChIP Buffer and sonicated to break nuclear membrane. After centrifugation at 9400 x g for 10 min, the supernatant was used for ChIP. ChIP was performed using CREB antibody (#9197, Cell Signaling) or phospho-CREB antibody (#9198, Cell Signaling) and ChIP-grade protein G magnetic beads (#9006, Cell Signaling). Normal rabbit IgG (#2729, Cell Signaling) was used as a negative control. ChIP products were eluted, and DNA was recovered through reverse crosslinking and purification. Specific binding of CREB to the *CREB*, *TNKS* and *KDM6A* promoter sites was quantified by qPCR. The following primers were used for qPCR: CREB (Forward: 5′-AGAAGCGGAGTGTTGGTGAG-3′, Reverse: 5′-TCCTCCTCCTGCTCCTCTTA-3′); TNKS BS1 (Forward: 5′-GGGGATGGCAGTCGGGAT-3′, Reverse: 5′-TTGTGCTGGTGGTACTGCAA-3′); TNKS BS2 (Forward: 5′-GACTAAGGGAAACAGAGCGG-3′, Reverse: 5′-GAGCTCAGAGAACAGCCAAGG-3′); KDM6A (Forward: 5′-CAGCACAACCTAACAGGAAGC-3′, Reverse: 5′-CGCCTCACGTGACCTTTGTT-3′); Negative control (Forward: 5′-ATCATGGCTTGGTGGGTTGT-3′, Reverse: 5′-AACCAAGAGACTGCAAGGCA-3′).

### RNA sequencing

Tumor spheroids were treated with cisplatin for 24 h, followed by total RNA isolation using the RNeasy Mini Kit (Qiagen, Germany). RNA quality was assessed with the Agilent 2100 Bioanalyzer (Agilent Technologies, Netherlands), and QuantSeq 3′ mRNA sequencing was outsourced to Ebiogen Inc. (Korea). Differentially expressed genes (DEGs) with a fold change of ≥ 2 were selected using the Excel-based ExDEGA software (Ebiogen, Korea).

### Regulatory network reconstruction and master regulator analysis

The Algorithm for the Reconstruction of Accurate Cellular Networks (ARACNe) was used to infer interactions between 826 transcription factors 42 and their target genes. A P-value of 0.05 was set for the mutual information (MI) threshold, with a data processing inequality (DPI) tolerance of 0%. Master Regulator Analysis (MRA) was applied to evaluate the significance of the overlap between the ARACNe-generated interactome and the signature genes. Signature genes were filtered based on an average fold-change ratio greater than 2 between cisplatin-sensitive and -resistant groups. ARACNe and MRA-Fisher's Exact Test (FET) analyses were conducted using the open-source geWorkbench platform (http://www.geworkbench.org), and the resulting networks were visualized in Cytoscape.

### Stable cell line generation

CREB shRNA (target sequence: 5′-GAGAGAGGTCCGTCTAATG-3′) 43 was cloned into the Tet-pLKO-puro vector for inducible shRNA expression (Plasmid #21915, Addgene, USA). To produce lentivirus, 293FT cells were transfected with Tet-pLKO-puro shMock or shCREB plasmids, along with pMD2.G and psPAX packaging plasmids. A549 cells were then incubated with the lentiviral supernatant for 3 days in the presence of 0.8 μg/ml polybrene, followed by selection with 2 μg/ml puromycin for 5 days.

### Mouse xenograft studies

Five-week-old male BALB/c nude mice were purchased from Orient Bio (Korea). A549 Tet-on-shCREB cells (5 × 10^6^), suspended in 100 μL PBS and mixed with 50 μL Matrigel (BD Biosciences, USA), were subcutaneously injected under isoflurane anesthesia (JW Pharmaceutical, Korea). When tumor volume reached approximately 100 mm³, mice were randomly divided into four groups. Two groups were given a doxycycline-containing diet to induce CREB knockdown, while the others were fed a normal diet. After 3 days, cisplatin (2.5 mg/kg) or saline was administered intraperitoneally twice weekly. Tumor sizes were measured twice a week, and the volume was calculated using the formula: V (mm³) = (length × width²) / 2. Mice were sacrificed before the tumor volume exceeded 1000 mm³.

### Immunohistochemistry

Xenograft tumors were removed and fixed in 4% paraformaldehyde, embedded in paraffin. Immunohistochemical staining performed with the automated instrument Discovery XT (Ventana Medical Systems, Inc., USA) using CREB (#9197, 1:2000), phospho-CREB antibody (#9198, 1:2000) KDM6A (#33510, 1:50), TNKS (GTX117417, 1:200). For immunohistochemical staining of tumor spheroids, tumor spheroids were treated with 400 μM pimonidazole (Hypoxyprobe^TM^-1) for 2 h, fixed in 4% paraformaldehyde, embedded in 2% low melting agarose, and then processed for paraffin embedding. Antibodies against pimonidazole (hypoxyprobe MAb1, clone 4.3.11.3, 1:400), Ki67 (ab15580, abcam, 1:1000), and cleaved caspase-3 (#9661, Cell signaling, 1:500) were employed in the staining of tumor spheroids.

### Public data analysis

All ChIP-seq data were obtained from the ChIP-Atlas database (http://chip-atlas.org/) and visualized using the Integrative Genome Viewer (IGV). The experimental IDs are as follows: CREB (SRX5576989), CTRC2 (SRX5576990), EP300 (SRX10976215), RNA polymerase II (DRX015225), H3K4me3 (SRX2410161), H3K27ac (SRX2409964) and ATAC-seq (SRX20495955). RNA expression data from lung adenocarcinoma, adjacent normal tissues, and healthy lung tissues were retrieved from The Cancer Genome Atlas (TCGA) and Genotype-Tissue Expression (GTEx) databases via the UCSC XENA platform (http://xena.ucsc.edu/). Protein expression data from lung adenocarcinoma and adjacent normal tissues were obtained from the Clinical Proteomic Tumor Analysis Consortium (CPTAC) database via cProSite (https://cprosite.ccr.cancer.gov/).

### Statistical analysis

Statistical analyses were carried out with GraphPad Prism 9. Data are represented as mean ± standard deviation (SD) or standard error of the mean (SEM). Student's t-test was used to compare two groups, and ANOVA was applied for comparisons among multiple groups. Significance was considered at *p* < 0.05.

## Results

### Tumor spheroids derived from various types of NSCLC cell lines exhibit a broad range of sensitivity to cisplatin

We first investigated whether our 3D culture method mimics *in vivo* tumor features, such as a gradient of O_2_ and nutrients, resulting is spatial distribution of proliferation rate of cancer cells depending on the distance from vascular supply 37. Previously, we reported that A549 and NCI-H460 tumor spheroids exhibit distinct growth characteristics, with A549 remaining non-growing and NCI-H460 undergoing continuous growth 44. Using these two models recapitulating slow-growing tumor and fast-growing tumor *in vivo*, we investigated whether our culture method reflects features of 3D tumor spheroids, including hypoxia, spatial distribution of cell death and proliferation rate 31.

**Figure 1:**
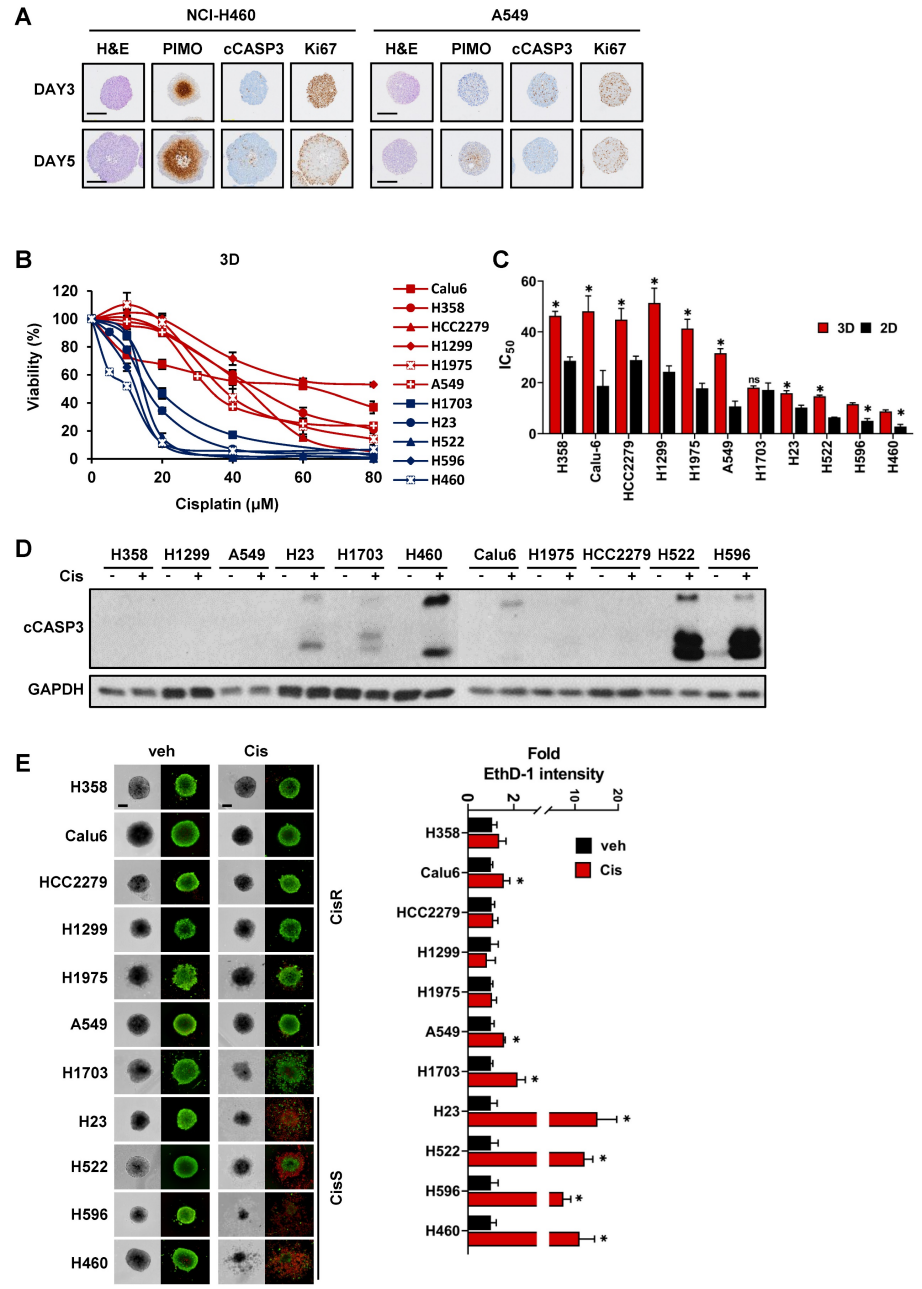
NSCLC cell-derived tumor spheroids exhibit a broad range of sensitivity to cisplatin. (A) Immunohistochemical staining of tumor spheroids generated from NCI-H460 and A549 cells. Tumor spheroids were fixed on day 3 and 5, stained with hematoxylin and eosin (H&E), pimonidazole (PIMO), cleaved caspase-3 (cCASP3), and Ki-67. Scale bar: 200 μm. (B) Dose-response curves of cisplatin in 11 tumor spheroids from NSCLC cells. Tumor spheroids were grown for 4 days and treated with various concentrations of cisplatin for 48 h. Cell viability was assessed by the ATP content (CellTiter-Glo 3D). Data represent the mean ± SEM of three independent experiments. (C) The IC_50_ values for each cell line cultured in 2D and 3D systems. *p < 0.05 vs. 2D. Data represent the mean ± SEM of three independent experiments. (D) Expression of the apoptotic marker in tumor spheroids exposed to cisplatin. Tumor spheroids were treated with 20 μM cisplatin for 48 h and the expression of cleaved caspase-3 was determined by western blotting. (E) Live/dead cell staining of tumor spheroids treated with cisplatin. Tumor spheroids were treated with 20 μM cisplatin for 48 h and then stained with calcein-AM (green, live cells) and ethidium homodimer-1 (EthD-1; red, dead cells). With the Operetta High Content Screening (HCS) system, images were acquired and EthD-1 intensity was analyzed. Scale bar: 200 μm. *p < 0.05 vs. veh. Data represent the mean ± SEM of three independent experiments.

Hematoxylin and eosin (H&E) staining was performed to examine the histological characteristics of the tumor spheroids (Fig. 1A). The hypoxic region, a feature of *in vivo* solid tumors and 3D tumor spheroids, was evaluated using pimonidazole, which covalently binds to the thiol groups of proteins in hypoxic cells at a partial oxygen pressure lower than 10 mmHg 45. Immunohistochemical staining for pimonidazole revealed hypoxic areas in the core region of the tumor spheroid. In growing H460 tumor spheroids, hypoxic regions appeared early and increased steadily over time, while in non-growing A549 tumor spheroids, weak hypoxic region appeared at day 5. Cleaved caspase-3-positive apoptotic cells increased over time in the H460 tumor spheroids, whereas apoptotic cells in the A549 tumor spheroids did not. Both A549 and H460 tumor spheroids showed homogeneous distribution of Ki67-positive cells, a marker of cell proliferation, on day 3. In H460 tumor spheroids grown for 5 days with a diameter over 500 μm, Ki67-positive cells were located in the outer layer, while the inner core exhibited necrosis, as described in previous 3D studies 31. Although A549 tumor spheroids did not display the same extent of changes as H460 tumor spheroids, possibly due to differences in the proliferation properties, they also exhibited 3D growth, such as a hypoxic core. These results suggest that our culture method replicates the characteristics of 3D tumor spheroids, including hypoxia and the distribution of proliferating cells due to oxygen and nutrient gradients 31.

Eleven NSCLC cell lines with various genetic backgrounds and growth properties were used in this study (Table S1) to determine sensitivity to cisplatin. Tumor spheroids derived from NSCLC cell lines displayed differential sensitivity to cisplatin and were divided into two groups: (i) cisplatin-sensitive and (ii) cisplatin-resistant in the range of 20 to 40 µM (Fig. 1B). Based on the IC_50_ for cisplatin, five cell lines (NCI-H1703, NCI-H23, NCI-H522, NCI-H596, and NCI-H460) were classified as cisplatin-sensitive (CisS, IC_50_ <20 µM), and six cell lines (A549, NCI-H1975, NCI-H1299, HCC2279, Calu-6 and NCI-H358) were classified as cisplatin-resistant (CisR, IC_50_ >20 µM) (Fig. 1C). Additionally, sensitivity to cisplatin was compared between the 3D and 2D cultures systems. The IC_50_ of cisplatin in 3D tumor spheroids was higher than that in 2D monolayer cultures, except for NCI-H1703 (Fig. 1B, C, and S1). To evaluate apoptotic cell death in the tumor spheroids, we analyzed the cleavage of caspase 3 and performed live/dead cell staining after cisplatin treatment (Fig. 1D, E). Consistent with the results of the viability assay, extensive apoptotic cell death was observed in CisS tumor spheroids. Although cisplatin-induced cell death in NCI-H1703 tumor spheroids was more than 2-fold (Fig. 1E), it was much lower than that in other sensitive tumor spheroids, and caspase-3 cleavage was minimal (Fig. 1D). Cisplatin inhibited the growth of NCI-H1703 tumor spheroids but was not sufficient to induce apoptotic cell death; therefore, we concluded that NCI-H1703 had intermediate sensitivity to cisplatin. In summary, cisplatin sensitivity is independent of the genetic background and growth properties of tumor spheroids.

### Reconstruction of transcriptional regulatory networks associated with cisplatin resistance

**Figure 2:**
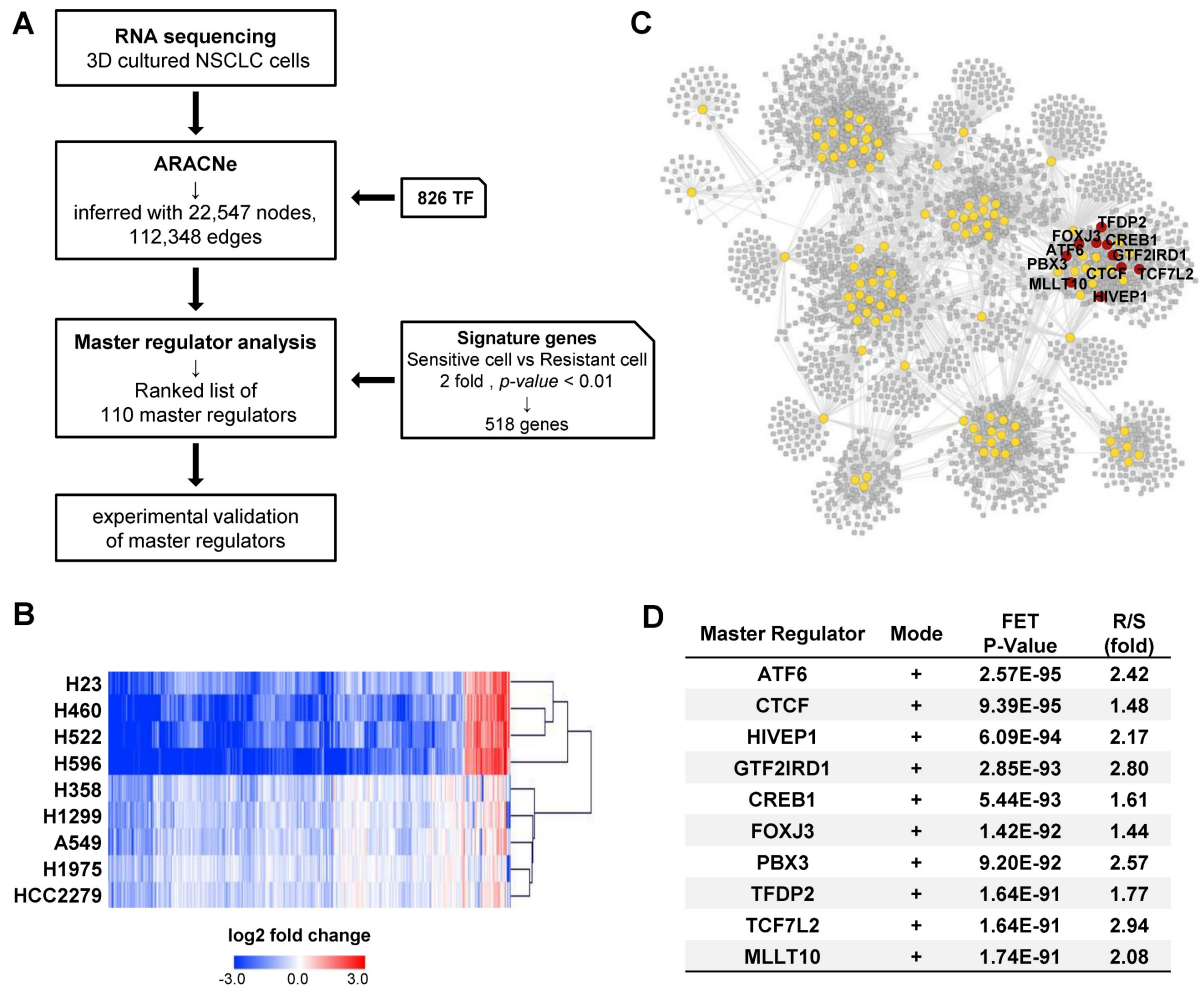
Reconstruction of gene regulatory networks to identify a master regulator. (A) Schematic overview of the workflow. Tumor spheroids derived from the 11 NSCLC cell lines were treated with cisplatin 20 μM for 24 h for RNA sequencing (RNA-seq). Using RNA-seq data, ARACNe and MRA were performed to identify the master regulators and their target genes. Then, 10 potential candidate transcription factors were functionally validated. (B) Heatmap showing the hierarchical clustering of the cisplatin-sensitive and -resistant cell lines and the fold-change values of 518 signature genes. The signature genes were filtered by the ratio of average fold-change in sensitive and resistant groups more than 2. (C) Network visualization of 110 master regulators which was predicted by ARACNe-MRA. The number of nodes and edges is 3,549 and 28,633, respectively. The yellow circles contain 110 master regulators and the red circle indicates top 10 master regulators. (D) List of top 10 master regulators ranked by the order of p-value. Plus mode means that the transcription factors are positively correlated with its target genes. R/S: average fold-change ratio of cisplatin to veh between resistant (R) and sensitive (S) groups.

To identify the key factors regulating cisplatin resistance, we performed RNA sequencing using tumor spheroids treated with cisplatin for 24 h and constructed a transcriptional network using an algorithm for the reconstruction of accurate cellular networks (ARACNe). The corresponding workflow is illustrated in Fig. 2A. ARACNe is a reverse engineering algorithm for inferring transcriptional regulatory networks based on mutual information, which is an information-theoretic measure of dependence among genes 42, 46. ARACNe predicted 112,348 interactions (edges) between 826 transcription factors (TFs) and 22,547 genes (nodes).

Next, we calculated the average fold-change in gene expression induced by cisplatin treatment in each CisS and CisR group and obtained a list of 668 differentially expressed genes with a ratio of more than 2 between the two groups. A heatmap of the 668 genes showed that the fold-change was higher in CisS cells than in CisR cells, and the number of downregulated genes was greater than that of upregulated genes (Fig. S2A). The NCI-H1703 was excluded because of their intermediate sensitivity to cisplatin. Calu-6 was also excluded because it clustered last in the resistant group, after NCI-H1703 (Fig. S2A). Therefore, four CisS (NCI-H23, NCI-H522, NCI-H596, and NCI-H460) and five CisR (A549, NCI-H1975, NCI-H1299, HCC2279, and NCI-H358) cell lines were used to extract signature genes, eventually identifying 518 signature genes that were clearly clustered into two distinct groups (Fig. 2B). Subsequently, 518 signature genes were used for master regulator analysis (MRA) to rank transcription factors whose regulons were enriched in the signature genes. MRA identified 110 master transcription factors that contribute to cisplatin sensitivity in NSCLC tumor spheroids. Because the entire network inferred by ARACNe was too complicated to display, we visualized the regulons of the 110 master transcription factors involved in 29,618 interactions using Cytoscape (Fig. 2C). We selected the top ten transcription factors ranked by p-values integrated using Fisher's method as candidate master regulators (MRs). All top 10 MRs were positive mode, indicating that the transcription factors were more active in the resistant group (Fig. 2D). Ten MRs were in the same network cluster (Fig. 2C); therefore, their target genes almost overlapped (Fig. S2B). We also found that a network was formed among the top 10 MRs, except for TFDP2, and that TCF7L2 may play a role as a common target gene for seven MRs, as well as one MR (Fig. S2C). Finally, we focused on the top ten MRs (ATF6, CTCF, HIVEP1, GTF2IRD1, CREB1, FOXJ3, PBX3, TFDP2, TCF7L2, and MLLT10).

### CREB is a master regulator of cisplatin sensitivity in NSCLC tumor spheroids

**Figure 3:**
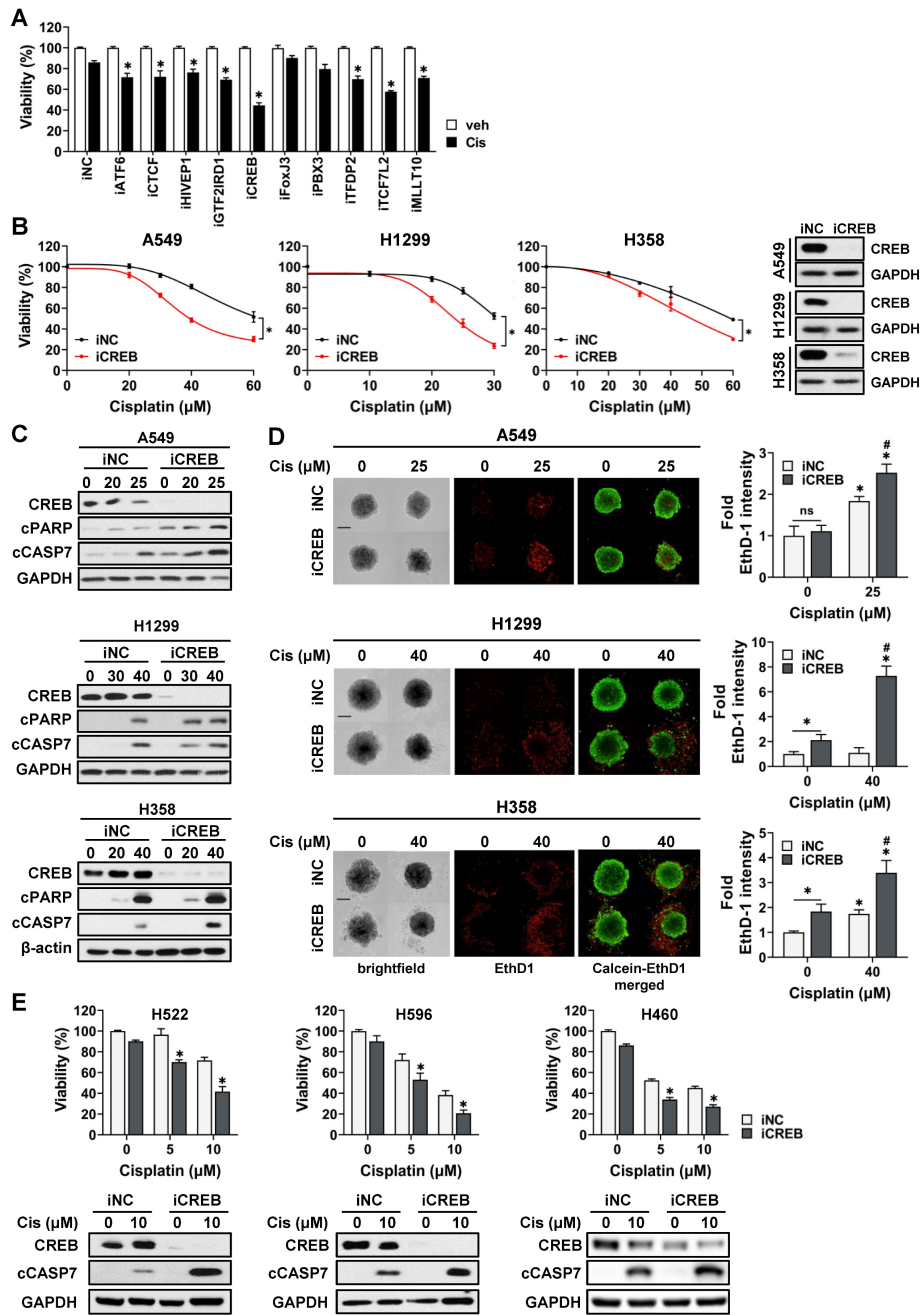
Inhibition of CREB enhances the sensitivity of NSCLC tumor spheroids to cisplatin. (A) Viability of CisR A549 tumor spheroids treated with cisplatin after siRNA knockdown of top 10 master regulators. siRNAs for each gene were transfected into A549 cells for 5 h and reseeded on ULA round bottom plates for the formation of tumor spheroids. After 3 days, tumor spheroids were treated with cisplatin (25 µM) for 48 h and cell viability was determined by measuring cellular ATP content. (B, C, D) The effect of CREB silencing on cisplatin sensitivity in three different resistant cell lines, A549, H1299, and H358. CREB knockdown tumor spheroids were treated with the indicated dose of cisplatin for 48 h and change of cisplatin sensitivity was evaluated via viability assay (B), western blot analysis (C), and live/dead staining (D). (E) The effect of CREB silencing on cisplatin sensitivity in three different sensitive cell lines, H522, H596, and H460. CREB knockdown tumor spheroids were treated with the indicated dose of cisplatin for 48 h and change of cisplatin sensitivity was evaluated via viability assay and western blot analysis. All data represent the mean ± SD of three independent experiments. *p < 0.05.

To confirm whether MRs were involved in cisplatin sensitivity, the viability of CisR A549 cells transfected with siRNAs targeting each MR was assessed in the presence of cisplatin. The knockdown of all transcription factors, except FoxJ3 and PBX3, attenuated the viability of tumor spheroids in response to cisplatin (Fig. 3A). Among these, CREB was found to be the most effective in increasing cisplatin sensitivity. To confirm the effect of CREB depletion on cisplatin sensitivity, we transiently transfected three CisR cell lines (A549, H1299, and H358) with CREB siRNA and treated them with varying concentrations of cisplatin. CREB knockdown decreased the viability of all CisR cells in the presence of cisplatin (Fig. 3B). Consistently, cisplatin increased the levels of cleaved caspase-7 and cleaved PARP, and the number of ethidium homodimer-1 (EthD-1)-positive dead cells increased in CREB-depleted cells treated with cisplatin (Fig. 3C, D). We also confirmed the effect of CREB knockdown on the sensitivity of CisS cells (H596, H522, and H460) to cisplatin and found that CREB inhibition was responsible for sensitization to cisplatin (Fig. 3E). Taken together, these results indicate that CREB is a master regulator of cisplatin sensitivity and that silencing of CREB sensitizes NSCLC cells to cisplatin-induced apoptosis.

### Cisplatin suppresses the expression of CREB via inhibiting CREB-autoregulation in cisplatin-sensitive tumor spheroids

**Figure 4:**
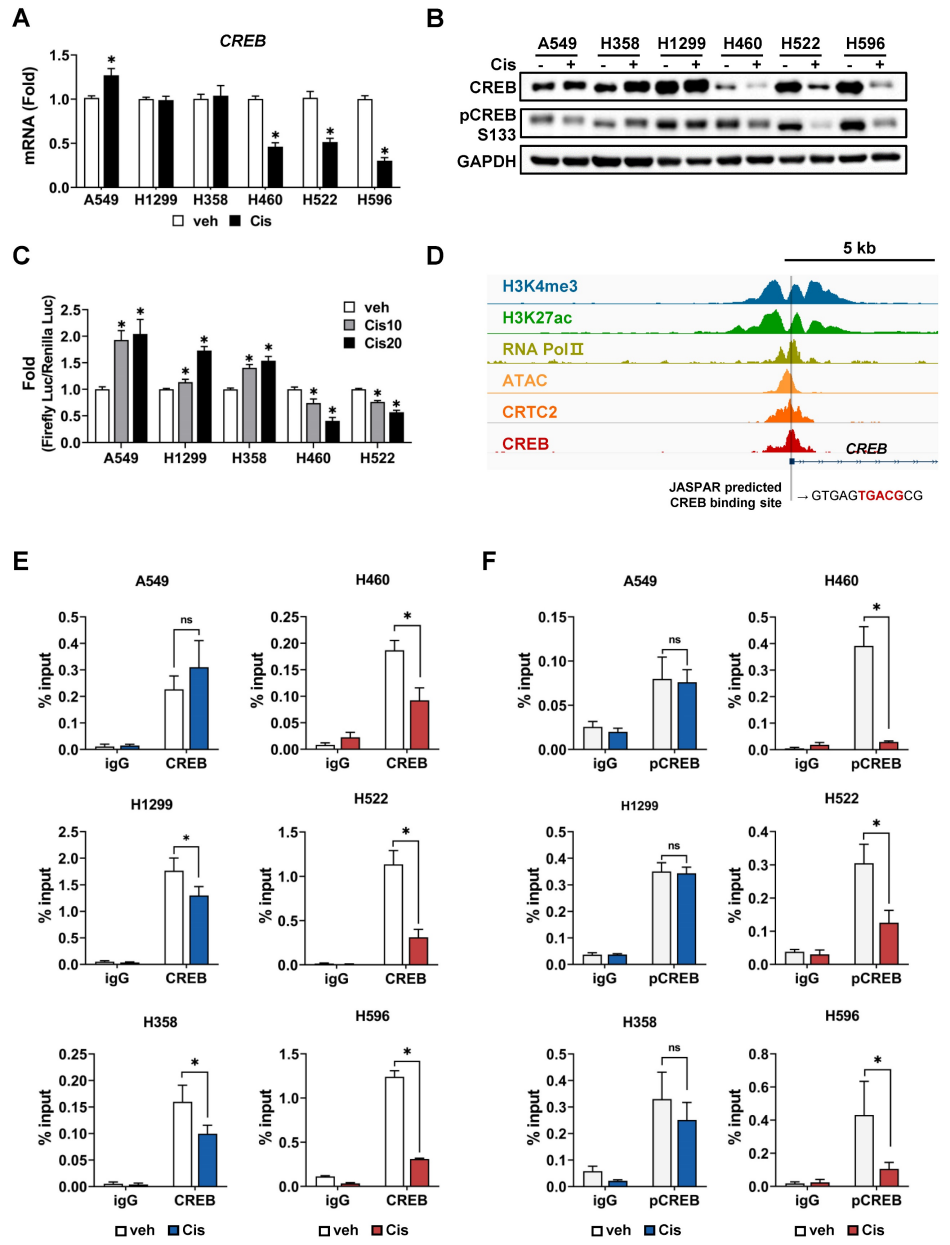
Phosphorylation of CREB and its transcriptional activation confers resistance to cisplatin. (A) Comparison of CREB mRNA levels between cisplatin-resistant and -sensitive cells. Tumor spheroids were treated with 20 μM cisplatin for 24 h, and mRNA levels were quantified using qPCR. Data are presented as fold-change normalized to GAPDH. Data represent the mean ± SEM of three independent experiments. *p < 0.05 vs. veh. (B) Comparison of CREB protein expression between cisplatin-resistant and -sensitive cells. Tumor spheroids were treated with 20 μM cisplatin for 24 h, and protein expression was evaluated by western blotting. (C) Comparison of CREB transcriptional activity between cisplatin-resistant and -sensitive cells. Cells were transfected with a CRE-luciferase reporter with pRL-CMV, followed by treatment with cisplatin. After 24 h, the luciferase activities were measured. Firefly luciferase activity was normalized to Renilla luciferase and expressed as the fold-change. Data represent the mean ± SEM of three independent experiments. *p < 0.05 vs. veh. (D) Analysis of CREB binding sites (BS) at the regulatory region of CREB. The Integrative Genomics Viewer browser shows the indicated ChIP-seq signals at the CREB regulatory regions. CREB binding sites predicted by JASPAR are represented by grey bars. (E-F) ChIP assay for binding of CREB (E) and pCREB S133 (F) to the regulatory region of CREB. Cisplatin-resistant (A549, H358, and H1299) and -sensitive (H460, H522, and H596) cells were treated with 20 μM cisplatin for 24 h, and a ChIP assay was conducted using antibodies against CREB, pCREB S133 or normal rabbit IgG and analyzed by qPCR. Data represent the mean ± SD. *p < 0.05 vs. veh.

Following cisplatin treatment, a difference was observed in the average fold-change in CREB expression between resistant and sensitive tumor spheroids (Fig. 2D). Changes in the mRNA and protein levels of CREB in response to cisplatin were measured in each tumor spheroid. Treatment with cisplatin at the dose of 20 µM did not reduce the expression of CREB in CisR cells (A549, H1299, and H358) but markedly decreased it in CisS cell lines (H460, H522, and H596) (Fig. 4A, B). In addition, the phosphorylation of CREB at Ser133, which activates CREB-mediated transcription, showed a similar trend (Fig. 4B). Cisplatin treatment induced significant changes in the mRNA and protein levels of CREB in CisS cells compared with those in CisR cells, suggesting that alterations in CREB expression may play a critical role in determining sensitivity to cisplatin. We examined the effects of cisplatin on CREB transcriptional activity and found that CRE-luc activity in CisS cells was inhibited by cisplatin, similar to the changes observed in mRNA and protein expression in cisplatin-treated CisS cells, whereas it increased in CisR cells (Fig. 4C).

CREB binds to the promoter region of its own gene, suggesting that it is positively auto-regulated 47, 48. This led us to hypothesize that the reduction of CREB, including phospho-CREB S133, and its transcriptional activity by cisplatin eventually results in decreased expression of the *CREB* gene. Using publicly available chromatin immunoprecipitation sequencing (ChIP-seq) data, we confirmed that the ChIP signal is abundant in the vicinity of 5'-UTR of *CREB*, and also identified a half-CRE motif in that region (Fig. 4D). To determine whether there was a difference in CREB occupancy depending on cisplatin sensitivity, we conducted a ChIP assay. Cisplatin treatment led to a substantial decrease in the binding of CREB and phospho-CREB S133 to their own regulatory regions in CisS cells, but not in CisR cells. The binding of phospho-CREB S133 was stronger than that of CREB in CisR cells following cisplatin treatment (Fig. 4E, F). These results imply that cisplatin inhibits the CREB binding to its own promoters and suppresses transcriptional activity in CisS cells, ultimately leading to reduced CREB expression. Thus, inhibiting CREB could be a strategy to reduce resistance to cisplatin.

### A functional approach to identify the potential target genes of CREB regulating cisplatin sensitivity

**Figure 5:**
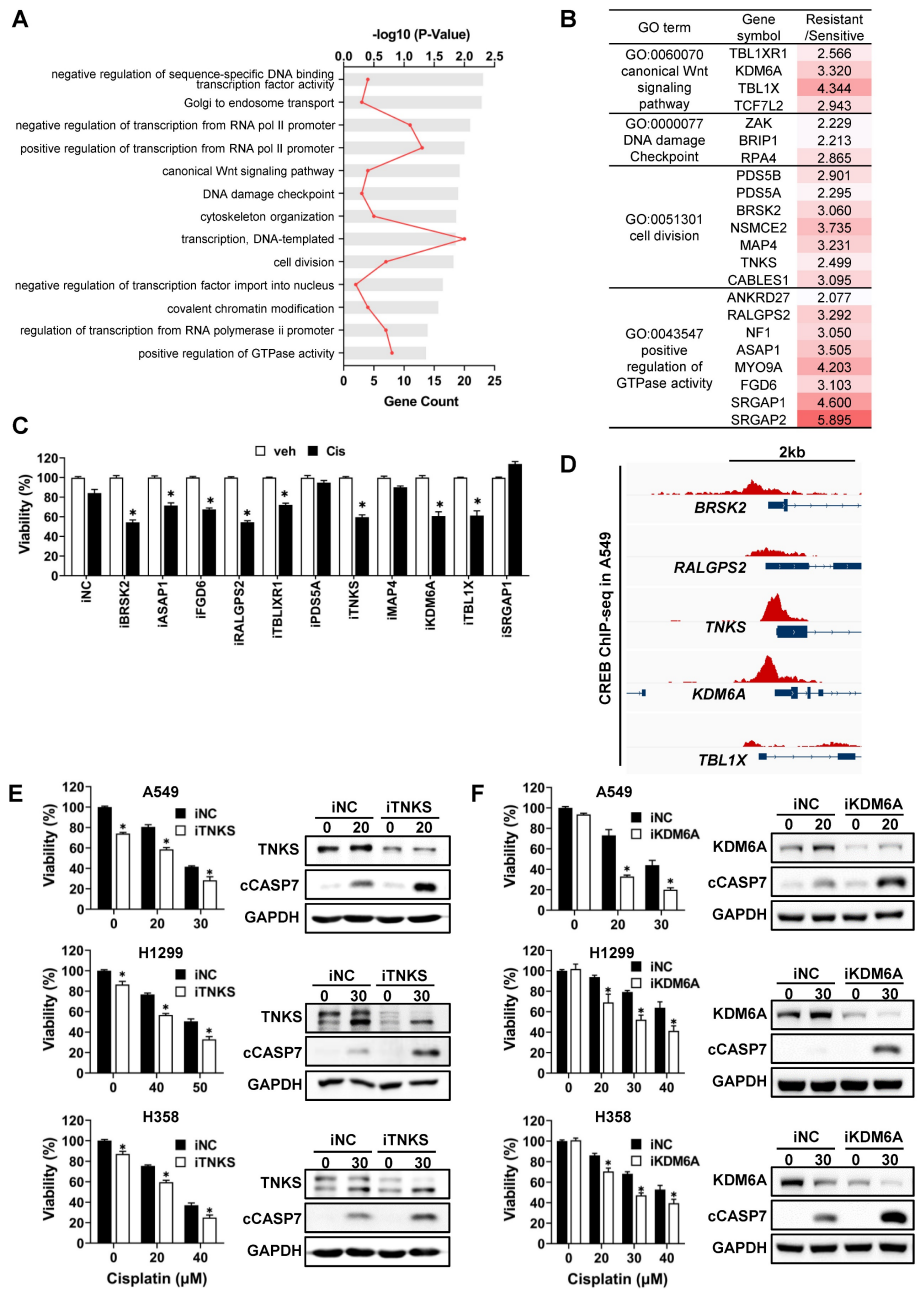
Functional approach to identify the potential target genes of CREB using gene ontology analysis. (A) Gene Ontology (GO) analysis of CREB target genes. The bar chart shows significantly enriched GO biological processes. GO enrichment analysis was performed using the DAVID database. (B) Summary of the 13 GO terms shown in (A), the list of genes that belong to indicated GO terms, and the ratios of average fold-change between cisplatin-sensitive and -resistant groups. (C) Viability of CisR A549 tumor spheroids treated with cisplatin after the siRNA knockdown of 11 target genes. siRNAs for each gene were transfected into A549 cells for 5 h and then reseeded on ULA round-bottom plates for tumor spheroid formation. After 3 days, the tumor spheroids were treated with cisplatin (25 µM) for 48 h, and cell viability was determined by measuring cellular ATP content. Data represent the mean ± SEM of three independent experiments. *p < 0.05 vs. veh. (D) Analysis of CREB enrichment at the regulatory regions of target genes. Using public CREB ChIP-seq data, CREB ChIP-seq signals at the regulatory regions of target genes were visualized using the Integrative Genomics Viewer. (E-F) Effect of TNKS and KDM6A silencing on cisplatin sensitivity in three different resistant cell lines, A549, H1299, and H358. TNKS (E) and KDM6A (F) silenced tumor spheroids were treated with the indicated dose of cisplatin for 48 h, and the change in cisplatin sensitivity was evaluated via viability assay and western blot analysis. Data represent the mean ± SEM of three independent experiments. *p < 0.05 vs. same dose of cisplatin-treated siNC.

Based on ARANCE and MRA, 109 genes were predicted to be potential targets of CREB from the 518 signature genes. To categorize target genes according to their functions, DAVID bioinformatics resources were used for Gene Ontology (GO) analysis. Biological process GO terms were largely classified into general transcriptional regulation, cell cycle, and others (Fig. 5A). We primarily focused on genes involved in cell proliferation and survival pathways that were activated in CisR cells. Among these, 11 genes were reported to play oncogenic roles in various tumors (Fig. 5B). To examine whether these 11 genes modulate cisplatin sensitivity in CisR A549 tumor spheroids, we measured cell viability following transfection with individual siRNAs and subsequent cisplatin treatment. Silencing of BR serine/threonine-protein kinase 2 (BRSK2), Tankyrase (TNKS), Ral GEF with PH domain and SH3 binding motif 2 (RALGPS2), lysine demethylase 6A (KDM6A), and transducin β-like 1 X-linked (TBL1X) expression decreased the viability of tumor spheroids in response to cisplatin by approximately 30% compared to the cisplatin-treated siNC (Fig. 5C). Given that the ChIP-seq signals for CREB obtained from the ChIP-Atlas were specifically enriched in the transcriptional regulatory regions of *TNKS* and *KDM6A* among the five candidate target genes (Fig. 5D), we focused on TNKS and KDM6A. Knockdown of TNKS and KDM6A sensitized CisR A549, H1299, and H358 tumor spheroids to cisplatin, resulting in reduced cell viability and increased levels of cleaved caspase-7 (Fig. 5E, F). Similarly, knockdown of BRSK2 led to increased sensitivity to cisplatin (Fig. S3A and S3B). Despite a weak CREB-ChIP-seq signal in the *BRSK2* promoter region (Fig. S3C), strong CREB enrichment was detected approximately 6-kb upstream of the *BRSK2* transcription initiation site, indicative of the presence of distal regulatory elements that may contribute to the regulation of *BRSK2* expression by CREB. In addition, the protein levels of TNKS, KDM6A, and BRSK2, which have been functionally identified to be involved in the regulation of cisplatin sensitivity, were significantly reduced after CREB knockdown followed by cisplatin treatment, compared to cisplatin-treated siNC (Fig. S4A). Consistent with the changes in protein levels, *TNKS* and *KDM6A* mRNA levels were reduced in all CisR tumor spheroids following CREB knockdown combined with cisplatin treatment (Fig. S4B). However, *BRSK2* mRNA levels were not altered by CREB knockdown with cisplatin treatment, except in H1299 tumor spheroids (Fig. S4B), suggesting that *BRSK2* is unlikely to be a direct transcriptional target of CREB. Taken together, these results highlight *TNKS* and *KDM6A* as direct CREB-regulated genes that contribute to cisplatin resistance.

### CREB regulates cisplatin sensitivity by directly targeting TNKS and KDM6A

**Figure 6:**
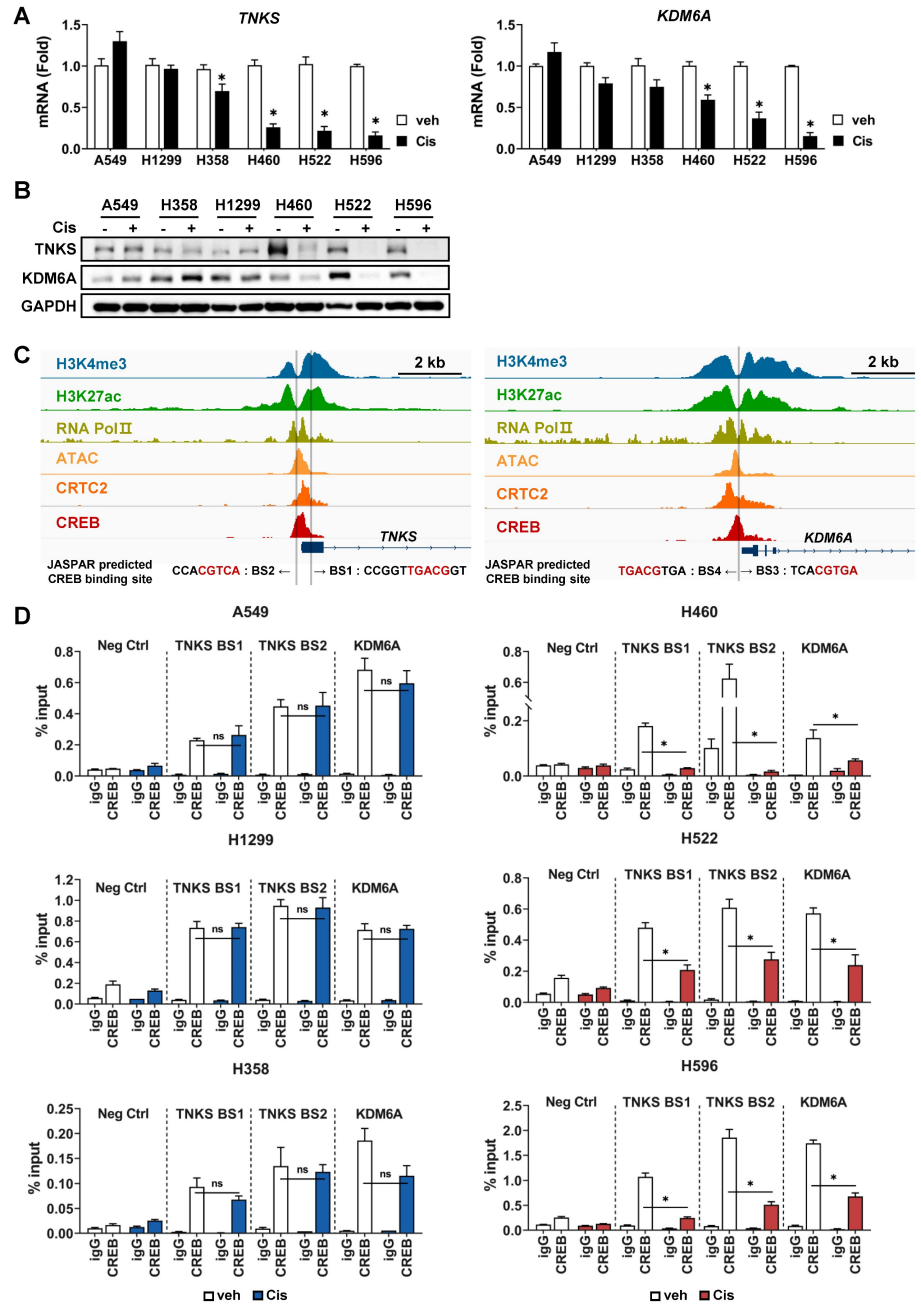

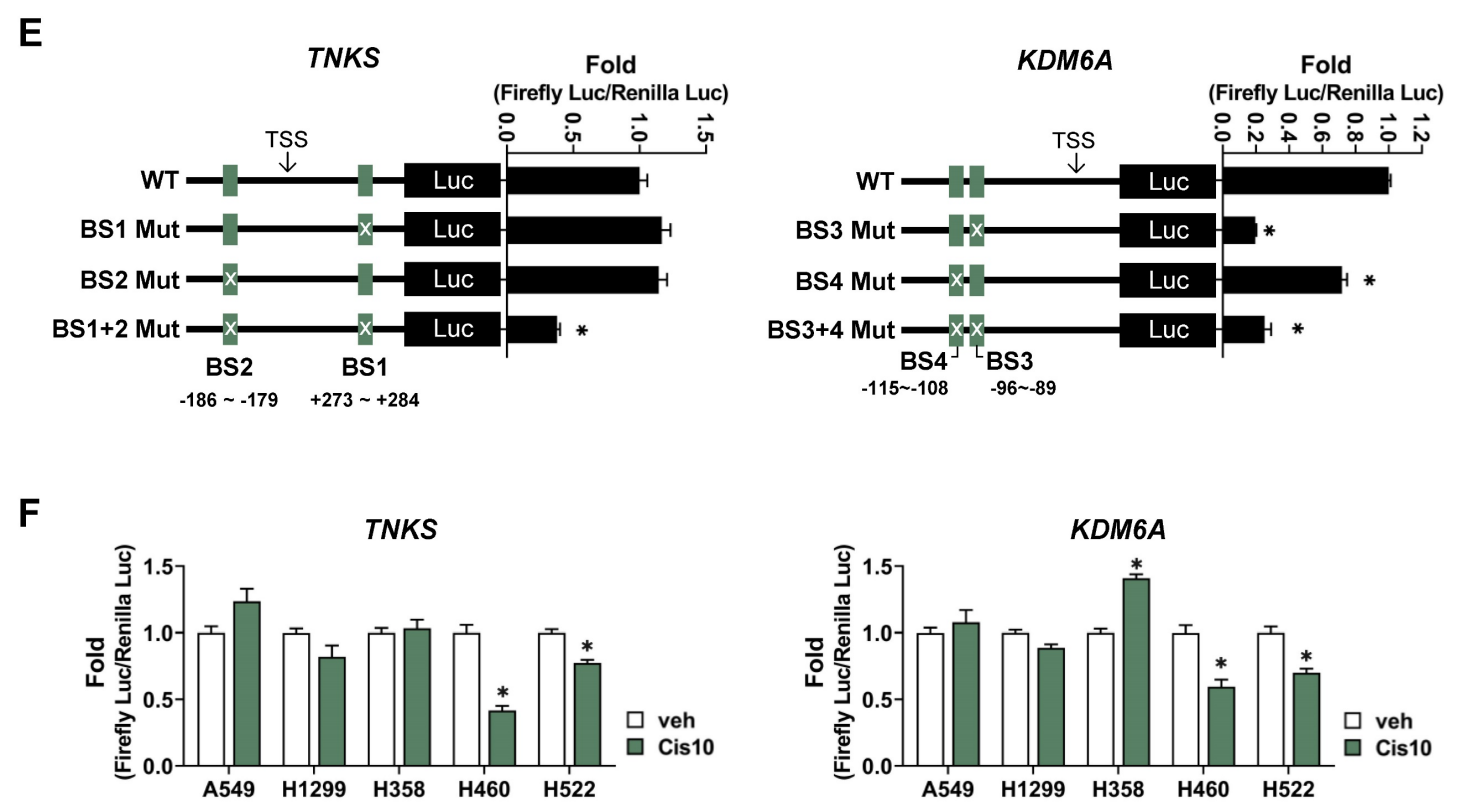
TNKS and KDM6A are transcriptional target of CREB and regulates cisplatin-sensitivity. (A) Comparison of TNKS and KDM6A mRNA levels between cisplatin-resistant and -sensitive cells. Tumor spheroids were treated with 20 μM cisplatin for 24 h, and the mRNA levels were quantified using qPCR. Data are presented as the fold-change normalized to GAPDH. Data represent the mean ± SEM of three independent experiments. *p < 0.05 vs. veh. (B) Comparison of TNKS and KDM6A protein expression between cisplatin-resistant and -sensitive cells. Tumor spheroids were treated with 20 μM cisplatin for 24 h, and protein expression was evaluated by western blotting. (C) Analysis of CREB binding sites (BS) at the regulatory regions of TNKS and KDM6A. The Integrative Genomics Viewer browser shows the ChIP-seq signals at the TNKS and KDM6A regulatory regions. CREB binding sites predicted by JASPAR are represented by grey bars. (D) ChIP assay for binding of CREB to regulatory regions of TNKS and KDM6A. Cisplatin-resistant (A549, H358, and H1299) and -sensitive (H460, H522, and H596) cells were treated with 20 μM cisplatin for 24 h, and a ChIP assay was conducted using antibodies against CREB or normal rabbit IgG and analyzed by qPCR. Data represent the mean ± SD. *p < 0.05 vs. veh. (E) A schematic representation of the wild-type (WT) and CREB-binding site mutant constructs corresponding to the regulatory regions of TNKS and KDM6A used in the luciferase assays. A549 cells were transfected with each construct along with the pRL-CMV vector for 24 h. Firefly luciferase activity was normalized to renilla luciferase activity and expressed as fold change relative to WT. Data represent the mean ± SEM of three independent experiments. p < 0.05 vs. WT. (F) Cisplatin-sensitive (H460, H522) and -resistant (A549, H1299 and H358) cells were transfected with WT constructs of TNKS and KDM6A for 24 h, followed by treatment with cisplatin for an additional 24 h. Luciferase activity was represented with fold change normalized to renilla luciferase activity. Data represent the mean ± SEM of three independent experiments. *p < 0.05 vs. veh.

To confirm whether the expression of *TNKS* and *KDM6A*, identified as potential CREB target genes, differs in response to cisplatin between CisS and CisR cells, we examined both mRNA and protein levels following cisplatin treatment. The *TNKS* and *KDM6A* mRNA and protein levels were reduced in cisplatin-treated CisS H460, H522, and H596 cells in response to cisplatin, whereas A549, H1299, and H358 cells exhibited no notable changes (Fig. 6A, B).

To validate whether *TNKS* and *KDM6A* are direct transcriptional target genes of CREB, the occupancy of CREB on the promoter region was examined using ChIP assay followed by qPCR analysis (ChIP-qPCR). The CREB ChIP-seq signal-enriched region shown in Fig. 5D was analyzed using JASPAR to predict CREB-binding sites. Two putative CREB-binding sites (BS1 and BS2) were identified in *TNKS*, one in exon 1 and the other in the promoter (Fig. 6C). Additionally, two CREB-binding sites (BS3 and BS4) were predicted in the promoter region of *KDM6A* (Fig. 6C).

Using public databases, we analyzed epigenetic markers for chromatin states, such as histone modifications and chromatin accessibility, and found that tri-methylated H3K4 (H3K4me3), acetylated H3K27 (H3K27ac), and ATAC-seq signals were enriched in the vicinity of the 5'-UTR or promoter region of *TNKS* and *KDM6A* (Fig. 6C). Furthermore, the ChIP-seq signals for RNA Pol II exhibited significant overlap with CREB binding sites, indicating that the CREB-binding region represents a transcriptionally active genomic locus. Therefore, ChIP-qPCR assays for CREB were performed using primers amplifying BS1, BS2 and BS3/4, respectively. The primer set for BS3/4 covers both sites, because BS3 and BS4 are only 11 base pairs apart. The results demonstrated that CREB bound to both the BS1 and BS2 of *TNKS* and BS3/4 site of *KDM6A* in all cell lines tested (Fig. 6D). In CisS H460, H522, and H596 cells, where CREB expression and transcriptional activity were reduced by cisplatin (Fig. 4), the occupancy of CREB on BS1 and BS2 of *TNKS* and BS3/4 of *KDM6A* cells significantly decreased following cisplatin treatment (Fig. 6D, right). In contrast, CREB binding remained largely unchanged after cisplatin treatment in CisR A549, H1299, and H358 cells (Fig. 6D, left).

Subsequently, we performed luciferase reporter assays to evaluate whether CREB binding to transcriptional regulatory regions of *TNKS* and *KDM6A* functionally affects transcriptional activity. Reporter constructs were designed to contain the intact CREB-binding site (wild-type), two single mutants, each disrupting one CREB sites, and a double mutant (Fig. 6E). For *TNKS*, mutation of either CREB-binding site individually did not significantly affect luciferase activity compared to the wild-type (Fig. 6E). However, simultaneous mutation of both sites resulted in a marked reduction in promoter activity by more than 60% (Fig. 6E), indicating that the two sites function in a compensatory manner to maintain gene expression, consistent with previous reports 49, 50. For *KDM6A*, mutation of BS4 resulted in a modest reduction in luciferase activity (~25%), while mutation of BS3 caused a more significant decrease (~70%) (Fig. 6E). Notably, the double mutant did not further reduce activity beyond that of the BS3 single mutant, suggesting that BS3 is the dominant functional site for CREB-mediated transcriptional regulation of *KDM6A*. Finally, to determine whether CREB-mediated transcriptional regulation of *TNKS* and *KDM6A* is modulated by cisplatin, we assessed promoter activity following cisplatin treatment. In CisS H460 and H522 cells, promoter activities of both *TNKS* and *KDM6A* were significantly decreased, whereas no change was observed in CisR A549, H1299, and H358 cells (Fig. 6F).

These findings indicate that *TNKS* and *KDM6A* are direct transcriptional targets of CREB and that alterations in CREB binding to the transcriptional regulatory regions of *TNKS* and *KDM6A* in response to cisplatin contribute to the observed differences in cisplatin sensitivity.

### CREB silencing combined with cisplatin significantly reduces *in vivo* tumor growth

**Figure 7:**
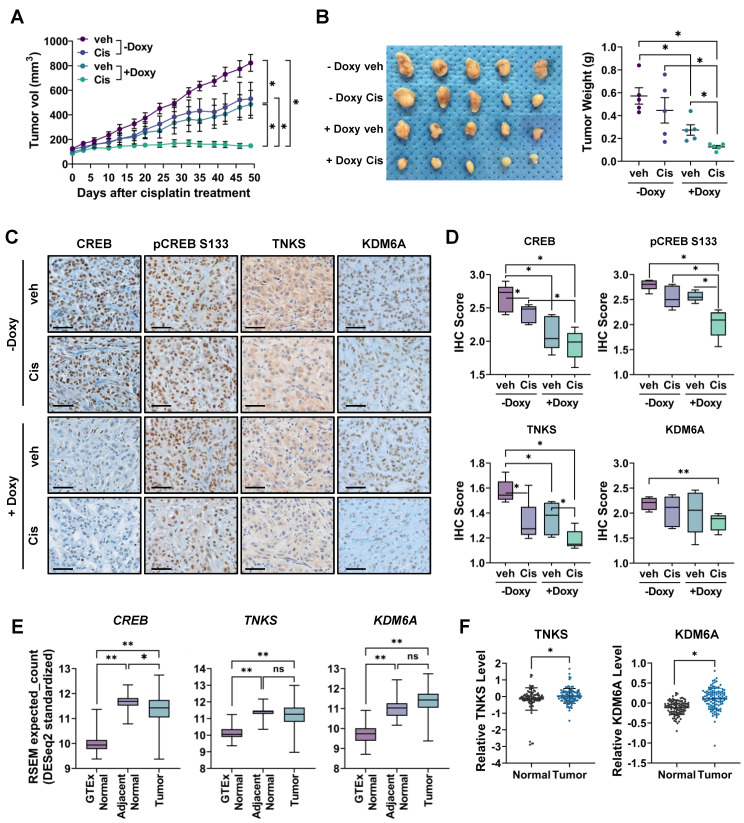
Inhibition of CREB enhances the antitumor effects of cisplatin in CisR A549 xenograft tumor. (A-B) Measurement of tumor volume (A) and weight (B). TetOn-shCREB A549 cells were injected subcutaneously into BALB/c nude mice. When tumor sizes reached approximately 100 mm³, mice were fed a doxycycline-containing diet for CREB knockdown and injected with either PBS or cisplatin (2.5 mg/kg) intraperitoneally twice a week. Tumor sizes were measured at the indicated times, and tumor volume was calculated as described in Materials and Methods. Data represent the mean ± SEM for tumor volume and mean ± SD for tumor weight. *p < 0.05. (C-D) Immunohistochemistry staining for CREB, TNKS, and KDM6A in tumor tissue. The staining intensity score was quantified using the IHC Profiler. Scale bar = 50 μm. (E) Differential mRNA expression profile of CREB, TNKS, and KDM6A among GTEx normal, adjacent normal, and lung adenocarcinoma tissues. Genotype-Tissue Expression (GTEx) normal lung tissues are from cancer-free individuals, while adjacent normal and tumor tissue data are from The Cancer Genome Atlas (TCGA). *p < 0.05, **p < 0.001. (F) Differential protein expression profile of TNKS and KDM6A between adjacent normal and lung adenocarcinoma tissue. Protein expression data from lung adenocarcinoma acquired from the Clinical Proteomic Tumor Analysis Consortium (CPTAC). *p < 0.05.

To determine whether CREB inhibition sensitizes CisR cells to cisplatin *in vivo*, we established a xenograft mouse model using A549 cells expressing a doxycycline-inducible shRNA that targets CREB. After establishing xenograft tumors, when the tumor volume reached approximately 100 mm^3^, one group of mice was fed a doxycycline-containing diet to induce CREB knockdown, whereas the control group was fed a normal diet. Tumors treated with cisplatin alone showed insignificant inhibition of tumor growth compared to the control group, whereas CREB knockdown alone significantly decreased both tumor volume and weight. Furthermore, cisplatin treatment combined with CREB knockdown dramatically suppressed tumor growth and tumor weight compared to the control, cisplatin, or CREB knockdown group (Fig. 7A, B).

Immunohistochemistry confirmed that CREB protein expression decreased in the tumors of mice fed a doxycycline-containing diet (Fig. 7C, D). Additionally, cisplatin treatment alone slightly reduced CREB expression, which was nearly completely reduced when combined with CREB knockdown. Phospho-CREB S133 expression was significantly reduced in the CREB knockdown group treated with cisplatin. Consistent with the *in vitro* results (Fig. S4), the expression levels of TNKS and KDM6A significantly decreased in tumors treated with both cisplatin and CREB knockdown.

Finally, we investigated the clinical implications of CREB, TNKS, and KDM6A expression in clinical samples using public datasets. *CREB*, *TNKS*, and *KDM6A* mRNA expression was significantly upregulated in NSCLC tissues compared to that in healthy lung tissues from Genotype-Tissue Expression (GTEx). However, compared to normal tissue adjacent to the tumor, *CREB* and *TNKS* mRNA expression was slightly downregulated, whereas *KDM6A* exhibited no significant difference (Fig. 7E). Using GTEx data instead of normal tissue adjacent to the tumor (NAT) for comparison with tumor tissues enables a clearer distinction of tumor-specific gene expression. NAT is morphologically normal, but in close proximity to the tumor, displays tumor-induced molecular changes, and possesses characteristics distinct from those of both healthy and tumor tissues 51. The elevated expression of *CREB* and its target genes in NAT compared to healthy lung tissue suggests that the tumor microenvironment may drive alterations in their expression. We also analyzed protein levels from the proteomic datasets of the Clinical Proteomic Tumor Analysis Consortium (CPTAC). The protein levels of TNKS and KDM6A were upregulated in tumor tissues compared to those in adjacent normal tissues (Fig. 7F), suggesting that these proteins could be promising targets for cancer treatment.

Taken together, our results demonstrate that targeting CREB sensitizes cisplatin-resistant NSCLC cells to cisplatin, suggesting that CREB inhibition combined with cisplatin is a potential strategy to overcome cisplatin resistance.

## Discussion

In this study, we identified CREB as a major transcriptional regulator of cisplatin resistance in NSCLC cells. CREB knockdown in cisplatin-resistant NSCLC cells increases cisplatin sensitivity *in vitro* and *in vivo*. Importantly, we identified *TNKS* and *KDM6A* as novel CREB target genes that directly bind to their transcriptional regulatory regions. Moreover, in CisS tumor spheroids, cisplatin treatment substantially decreased the expression of CREB mRNA and protein (Fig. 4A and 4B), and decreased the levels of TNKS and KDM6A proteins (Fig. 6A and 6B), resulting in a substantial increase in apoptotic cell death (Fig. 1D and 1E). In contrast, CisR tumor spheroids maintained the expression of CREB and its downstream targets TNKS and KDM6A, as well as cell survival in response to cisplatin. Upon CREB knockdown, the expression of TNKS and KDM6A was abolished by cisplatin treatment (Fig. S4), thereby inducing a response similar to that observed in cisplatin-sensitive cells.

CREB has been implicated in chemotherapy resistance mechanisms across various cancer types. In particular, its role in platinum-based chemotherapy resistance has been highlighted in various studies. CREB has been shown to mediate cathepsin L-induced cisplatin resistance in A549 cells, promoting cell survival under cisplatin treatment 52. In triple-negative breast cancer, CREB was identified as a key transcription factor regulating RAS protein activator-like 2 (RASAL2), a crucial factor associated with resistance to platinum-based chemotherapy 53. In ovarian cancer, inhibition of CREB activity by H89, a PKA inhibitor, was reported to sensitize platinum-resistant cells to cisplatin 54. Beyond platinum-based therapies, CREB has also been associated with resistance to other chemotherapeutic agents, doxorubicin and gemcitabine 55, 56. Collectively, these studies suggest that CREB-mediated resistance mechanisms may not be limited to NSCLC but could also play a role in chemoresistance across different cancer types.

Cisplatin, a cornerstone chemotherapeutic agent, is effective against various types of cancers. However, innate or acquired resistance to cisplatin significantly limits its therapeutic efficacy. Cisplatin resistance occurs through a variety of complex mechanisms, including decreased intracellular accumulation, increased inactivation, increased repair of damaged DNA, and inactivated of apoptotic signals 7. Ultimately, cisplatin resistance occurs because the drug fails to effectively induce cell death, and consequently contributes to tumor recurrence and metastasis, leading to poor patient outcomes. Understanding the mechanisms underlying cisplatin resistance will contribute to the development of therapeutic approaches for overcoming or preventing resistance.

Targeting transcription factors offers several advantages in overcoming drug resistance. When a specific pathway is blocked by a drug, cancer cells bypass or activate alternative pathways to evade cell death, thereby inducing drug resistance. However, modulating transcription factors that directly regulate the expression of multiple essential genes at the final step of signaling pathways is likely to exhibit a better efficacy and a lower chance of developing resistance, thereby providing a potential strategy for overcoming drug resistance 57. CREB is a transcription factor involved in the initiation and progression of many types of tumors by regulating various cellular processes, including proliferation, apoptosis, metabolism, and immune surveillance 58. Several CREB inhibitors, such as 666-15, disrupt the interaction between phospho-CREB S133 and its coactivator CBP, thereby inhibiting CREB transcriptional activity and transcription of its target genes 59-61.

We identified *TNKS* and *KDM6A* as novel CREB target genes and demonstrated their involvement in the regulation of cisplatin sensitivity. Results from ChIP and luciferase reporter assays provide direct evidence that CREB regulates transcription of *TNKS* and *KDM6A* through multiple binding sites (Fig.6). For *TNKS*, mutation of either CREB-binding site alone did not significantly affect transcriptional activity of CREB, whereas simultaneous mutation of both sites led to a marked reduction. This result is consistent with the homotypic cluster model, in which clustered transcription factor binding sites can compensate for each other, so that disrupting a single site has little or no effect on transcription unless multiple sites are disrupted simultaneously 49, 62. A similar phenomenon has been observed in the human *COX-2* promoter, where individual mutations of two NFAT-binding sites had minimal effects, but simultaneous mutation of both sites almost completely abolishes transcriptional activity 50. In *KDM6A*, although both CREB-binding sites were functional, the significant decrease in transcriptional activity observed upon BS3 mutation and the absence of an additive effect in the double mutant, suggests that BS3 serves as the primary regulatory site.

TNKS is a poly-ADP-ribose polymerase enzyme that regulates key cellular pathways, particularly Wnt/β-catenin signaling. TNKS promotes the degradation of AXIN proteins, leading to β-catenin stabilization and the activation of Wnt signaling, which in turn drives cell proliferation and survival 63. TNKS expression is upregulated in lung cancer and correlated with poor prognosis 64, 65. In addition, inhibitors targeting TNKS are being explored as potential therapies strategy to suppress tumor growth driven by aberrant Wnt/β-catenin signaling 66-68.

KDM6A is a lysine-specific demethylase that plays a crucial role in gene regulation by removing methyl groups from histone H3 at lysine 27 (H3K27), thereby activating transcription 69. KDM6A often functions as a tumor suppressor, particularly in bladder cancer, where mutations can lead to loss of function. These mutations contribute to tumorigenesis by promoting immune escape and enhancing the metastatic potential 70, 71. However, KDM6A can act as a tumor promoter in certain cancers, such as breast 72, colorectal 73, and lung cancer 74, 75. In NSCLC, KDM6A overexpression is associated with a poor prognosis, and KDM6A knockdown significantly reduces tumorigenic properties 74. Additionally, KDM6A overexpression enhances chemoresistance in NSCLC stem cells and is associated with increased tumor recurrence following cisplatin treatment 75. The results of the present study provide a rationale for the combination treatment of cisplatin and CREB inhibition for chemoresistant NSCLCs, with the identification of CREB target genes, *KDM6A* and *TNKS*, for the first time.

In this study, cisplatin-induced change in CREB expression were identified as a key event in the regulation of cisplatin sensitivity. To investigate the mechanism by which CREB is differentially regulated, we compared the activity of signaling pathways related to cell survival, death, and DNA damage response (Fig. S5). Cisplatin treatment did not affect the phosphorylation of protein kinase A (PKA), the kinase directly phosphorylates CREB 12 . The activity of ATR and Chk1, kinases phosphorylated in response to cisplatin-induced DNA damage 76, showed no notable differences between the CisR and CisS. Similarly, the cell survival regulator Akt also exhibited no significant differences between the two groups.

Among MAP kinase JNK, p38 and Erk1/2, which are key components of the MAPK pathway involved in enhancing CREB S133 phosphorylation 77, JNK and p38 were generally more activated in the CisS groups (Fig.S5). However, their activation does not correlate with S133 phosphorylation of CREB by cisplatin (Fig. 4B). Instead, their activation appears to be associated with pro-apoptotic signaling 78, consistent with the enhanced sensitivity of CisS cells to cisplatin. The inhibitory role of JNK in the regulation of CREB has been suggested. Ro31-8220, a small molecule with anti-melanogenic activity, activated JNK, which phosphorylated CRTC3, a CREB coactivator, independent of CREB Ser133 phosphorylation, preventing its nuclear translocation and thereby inhibiting CREB activation 79. Given that CRTCs serve as coactivators, independently of Ser133 phosphorylation of CREB 24, their inhibition by activated JNK in CisS tumor spheroids may contribute to increased cisplatin sensitivity. Similarly, p38 has also been shown to regulate CREB indirectly. p38 inhibited the PKD1 activation, a kinase that phosphorylates CREB at Ser133, there by promoting apoptosis in response to selenite in colorectal cancer cells 80. These findings align with our observation that CREB phosphorylation was reduced in CisS cells along with increased p38 activation. This suggests that p38 may suppress Ser133 phosphorylation of CREB by interfering with its upstream kinase and thus inhibit CREB autoregulation, ultimately resulting in reduced CREB expression and thus increased cisplatin sensitivity in CisS tumor spheroids.

STAT3 phosphorylation displayed one of the most notable differences between the CisS and CisR groups (Fig. S5). STAT3 is activated through phosphorylation at tyrosine 705 in response to various cytokines and growth factors, inducing the expression of a variety of genes associated with cell growth, survival, and motility. Specifically, sensitivity to cisplatin has been reported to increase when STAT3 is inhibited in various cancers 81, which is consistent with our findings. However, the relationship between STAT3 and CREB remains poorly understood. It has been reported that CREB acts as an upstream regulator of STAT3 during the differentiation of mesenchymal stem cells induced by nanosecond pulsed electric fields 82. Conversely, in inflammatory breast cancer, cAMP-PKA-CREB signaling has been suggested to acts downstream of JAK/STAT3 83. In NSCLC cells, STAT3 inhibition did not lead to consistent changes in CREB expression (Fig. S6). These findings suggest that CREB expression is regulated by alternative pathways independent of STAT3. Among upstream kinase we tested, the stress-responsive kinases p38, as described above, may provide a possible explanation for the differential changes in CREB expression in response to cisplatin between the CisR and CisS groups.

Taken altogether, our study demonstrated that cisplatin-induced CREB expression contributes to the differential cellular response to cisplatin in NSCLC, and CREB inhibition effectively sensitize NSCLC tumor spheroids and tumors to cisplatin. Thus, targeting CREB represents a potential therapeutic strategy in combination with chemotherapy to improve clinical outcomes, particularly in patients with platinum resistance.

## Supplementary Material

Supplementary figures and table.

## Abbreviations

NSCLC: non-small cell lung cancer
CREB: cAMP response element-binding protein 1
bZIP: basic leucine zipper
CRE: cAMP response element
PKA: protein kinase A
MAPK: mitogen-activated protein kinase
CaMK: calcium/calmodulin-dependent protein kinases
Akt: Protein kinase B
CBP: CREB-binding protein
CRTCs: CREB-regulated transcriptional coactivators
EP300: E1A binding protein p300
ARACNe: algorithm for the reconstruction of accurate cellular networks
MRA: master regulator analysis
ATF6: activating transcription factor 6
CTCF: CCCTC-binding factor
HIVEP1: human immunodeficiency virus type 1 enhancer-binding protein 1
GTF2IRD1: general transcription factor II-I repeat domain-containing protein 1
FOXJ3: forkhead Box J3
PBX3: Pre-B-cell leukemia transcription factor 3
TFDP2: Transcription factor Dp-2
TCF7L2: transcription factor 7-like 2
MLLT10: mixed-lineage leukemia translocated to 10
BRSK2: BR serine/threonine-protein kinase 2
TNKS: tankyrase
RALGPS2: Ral GEF with PH domain and SH3 binding motif 2
KDM6A: lysine demethylase 6A
TBL1X: transducin beta-like 1 X-linked
STAT3: signal transducer and activator of transcription 3
ATR: ataxia telangiectasia and Rad3-related protein
Chk1: checkpoint kinase 1
JNK: c-Jun N-terminal kinases
