# Supplementary material for: CREB Regulates Cisplatin Resistance by Targeting TNKS and KDM6A in NSCLC cell-Derived Tumor Spheroid
Source: Int J Biol Sci. 2025 Jul 28;21(11):4851–71. doi: 10.7150/ijbs.109419 (PMC12374816; doi:10.7150/ijbs.109419)
Supplement: Supplementary file 1 — Supplementary figures and table. [file ijbsv21p4851s1.pdf]

Supplementary materials

CREB Regulates Cisplatin Resistance by Targeting TNKS and KDM6A in NSCLC cell-Derived Tumor Spheroids

Ji Hae Lee[a,b], Youngjoo Kwon [b], Kyungsil Yoon[a]*

[a] Cancer Metastasis Branch, National Cancer Center, Goyang 10408, Korea

[b] College of Pharmacy, Graduate School of Pharmaceutical Sciences, Ewha Womans University, Seoul 03760, Korea

*Correspondence: kyoon@ncc.re.kr

Supplementary Table 1. Characteristics of the 11 NSCLC cell lines used in this study

Cell line	Histology	EGFR	RAS	TP53	Growth property
A549	Adenocarcinoma	WT	K-RAS G12S	WT	non
NCI-H1299	Large cell carcinoma	WT	N-RAS Q61K	Null	growing
NCI-H358	Adenocarcinoma	WT	K-RAS G12C	Null	growing
NCI-H1975	Adenocarcinoma	L858R, T790M	WT	R273H	growing
HCC2279	Adenosquamous Carcinoma	del(E746-A750)	WT	Y234C	non
Calu-6	Adenocarcinoma	WT	K-RAS Q61K	R196Ter	growing
NCI-H1703	Squamous Cell Carcinoma	WT	WT	c.919+1G>T	growing
NCI-H460	Large cell carcinoma	WT	K-RAS Q61H	WT	growing
NCI-H522	Adenocarcinoma	WT	WT	P191fsTer56	growing
NCI-H596	Adenosquamouse carcinoma	WT	WT	G245C	non
NCI-H23	Adenocarcinoma	WT	K-RAS G12C	M246I	growing

Abbreviations: WT, wild-type; Ter, termination; fs, frameshift

Cell line information accessed from DepMap portal website: https://depmap.org/portal

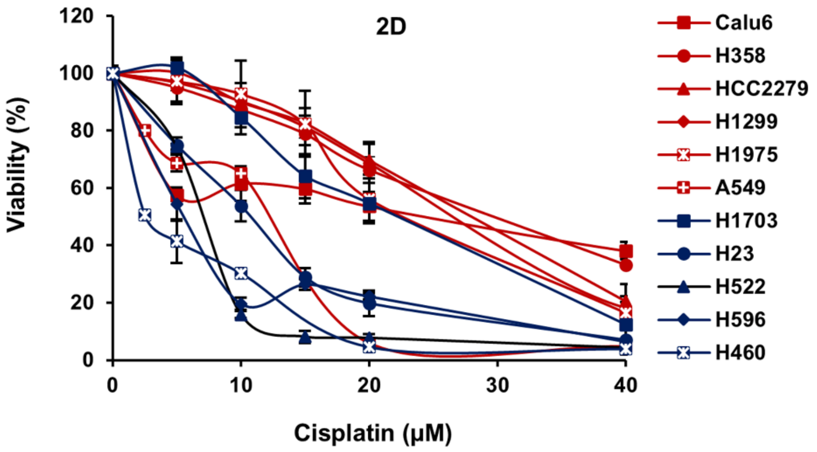

Supplementary Figure 1. Dose-response curves of cisplatin in 2D cultured NSCLC cells. Cells were treated with various concentrations of cisplatin for 48 h. Cell viability was assessed by the ATP content (CellTiter-Glo 2.0). Data represent the mean ± SEM of three independent experiments.

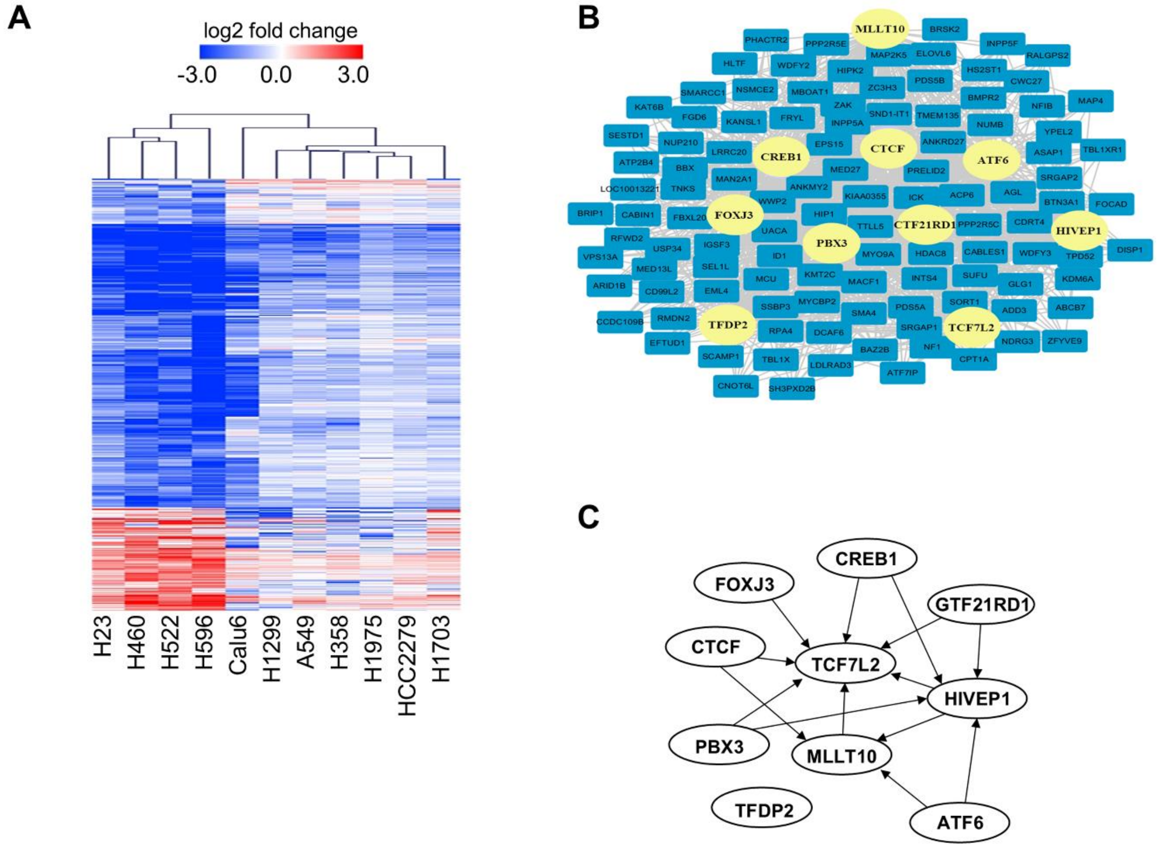

Supplementary Figure 2. Transcriptional regulatory networks associated with cisplatin resistance. (A) A heatmap showing the hierarchical clustering of the cisplatin-sensitive and -resistant cell lines and the fold-change values of 668 genes. (B) The network consists of the top 10 master regulators and their target genes. Yellow nodes represent the master regulators. (C) A schematic representation of transcriptional network between top 10 master regulators.

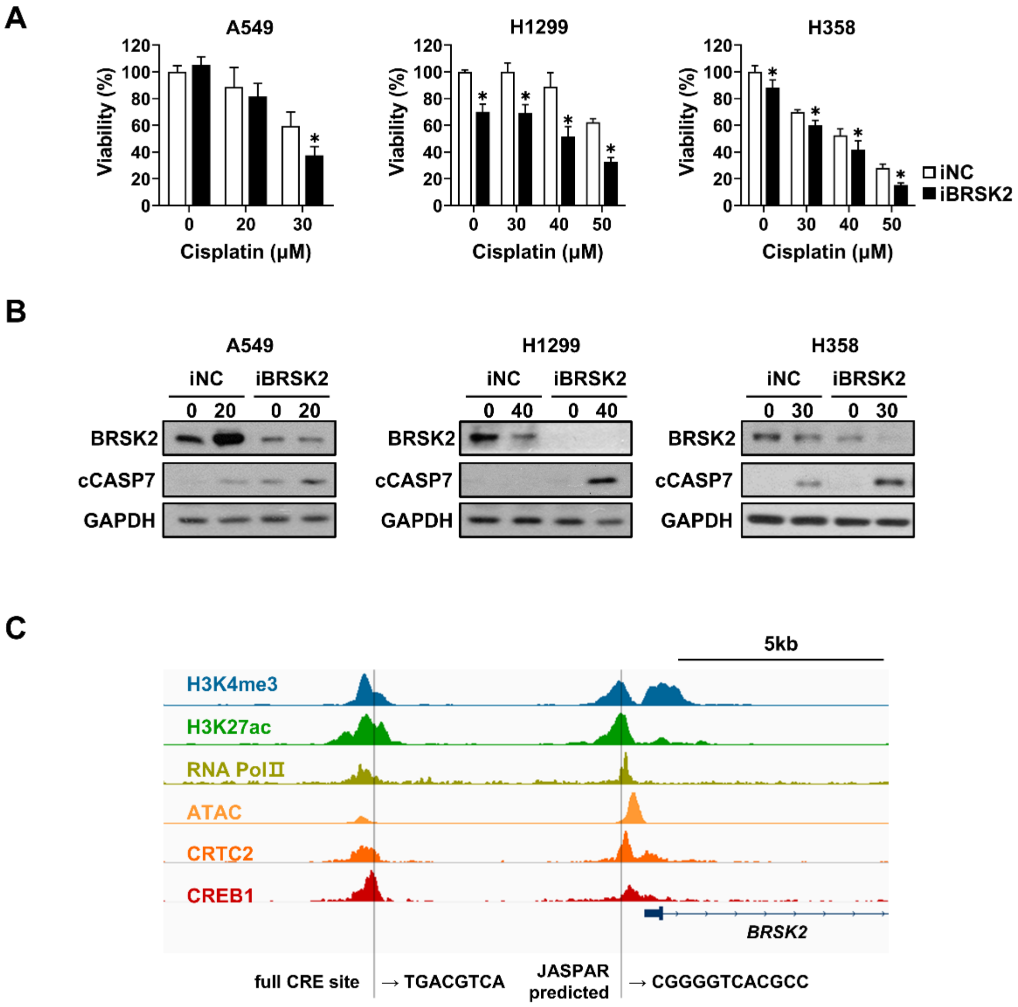

Supplementary Figure 3. Inhibition of BRSK2 enhances the cisplatin sensitivity. (A, B) The effect of BRSK2 silencing on cisplatin sensitivity was analyzed in three cisplatin-resistant cell lines (A549, H1299, and H358). Tumor spheroids with BRSK2 silencing were treated with the indicated dose of cisplatin for 48 h, and changes in cisplatin sensitivity were assessed via viability assay (A) and western blot analysis (B). (C) Analysis of CREB binding sites in the regulatory regions of *BRSK2*. The Integrative Genomics Viewer browser displays ChIP-seq signals in the regulatory regions of *BRSK2*. CREB binding sites predicted by JASPAR are indicated by grey bars.

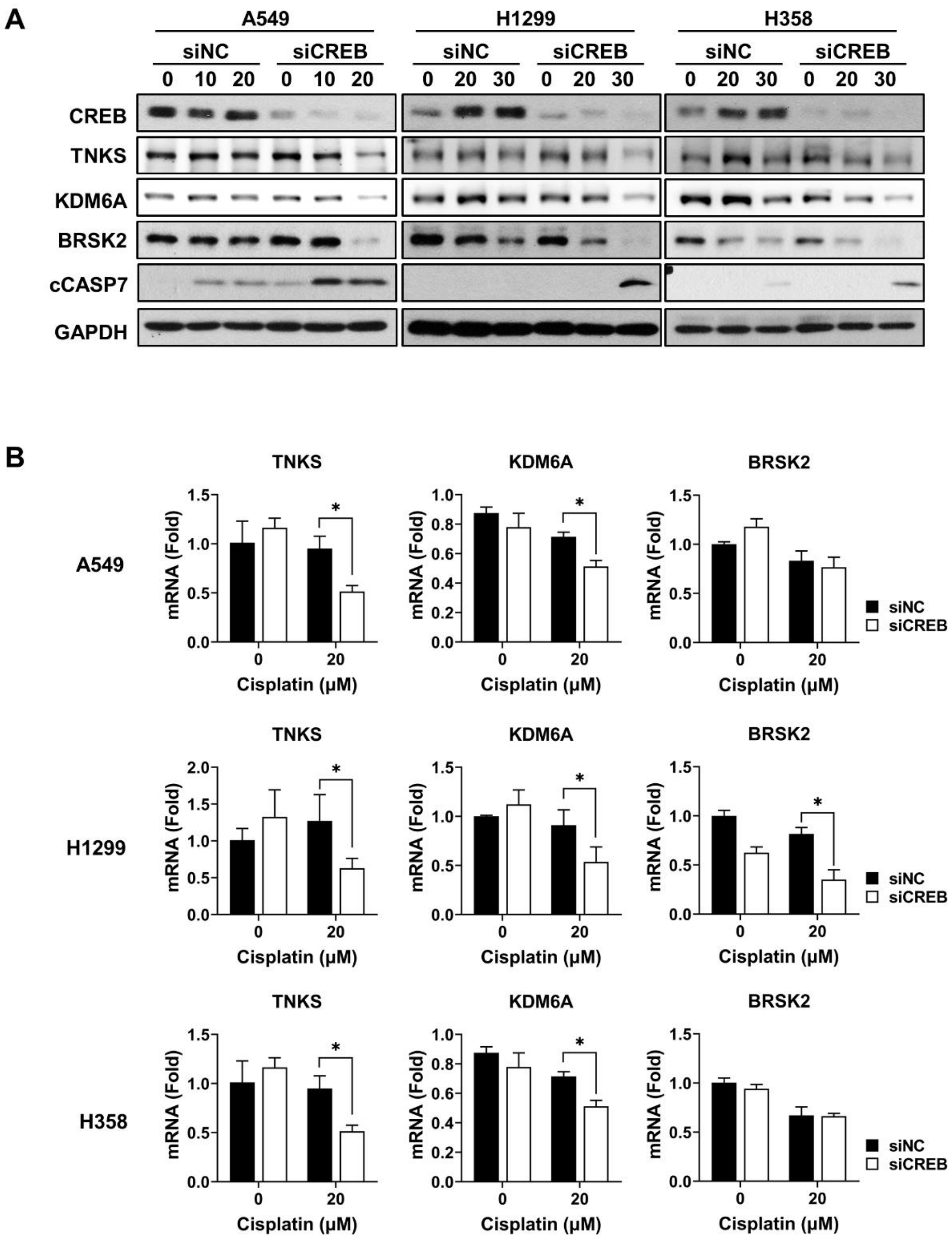

Supplementary Figure 4. CREB knockdown combined with cisplatin treatment reduces the expression of TNKS, KDM6A, and BRSK2. Tumor spheroids with CREB knockdown were treated with the indicated dose of cisplatin for 48 h, and changes in the protein (A) and mRNA (B) expression levels of CREB target genes were evaluated by western blotting and qPCR, respectively.

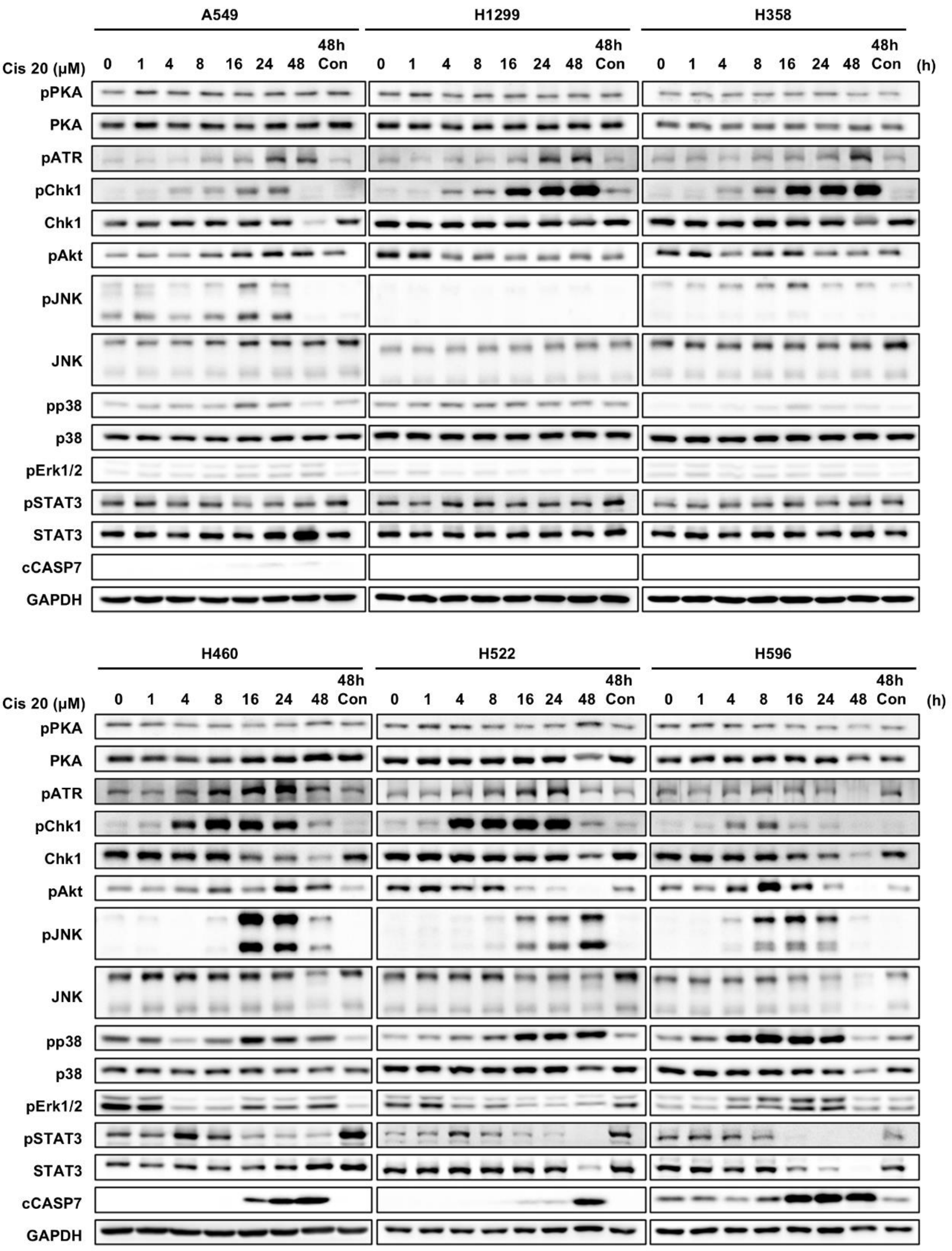

Supplementary Figure 5. Comparison of signaling pathways between cisplatin-resistant and cisplatin-sensitive tumor spheroids. Tumor spheroids were treated with 20 μM cisplatin for the indicated time. Protein expression associated with cell survival, cell death, and DNA damage response was analyzed by western blotting.

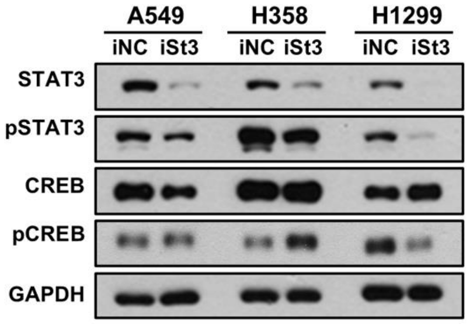

Supplementary Figure 6. STAT3 is not involved in CREB expression. Cells were transfected with siRNA targeting STAT3 for 48 h, and the expression levels of CREB and pCREB were analyzed by western blotting.
